# Immunogenicity risk assessment of peptide-related impurities identified in generic teriparatide products

**DOI:** 10.3389/fimmu.2025.1730346

**Published:** 2025-12-08

**Authors:** Aimee E. Mattei, Brian J. Roberts, Sandra Lelias, Shah Miah, Kristina E. Howard, James L. Weaver, Daniela Verthelyi, Eric S. Pang, Katie Edwards, Anne S. De Groot

**Affiliations:** 1 EpiVax Inc, Providence, RI, United States; 2 Division of Applied Regulatory Sciences, Office of Clinical Pharmacology, Office of Translational Sciences, Center for Drug Evaluation and Research, U. S. Food and Drug Administration, Silver Spring, MD, United States; 3 Office of Pharmaceutical Quality Research-Division IV, Center for Drug Evaluation and Research, U.S. Food and Drug Administration, Silver Spring, MD, United States; 4 Division of Therapeutic Performance I, Office of Research and Standards, Office of Generic Drugs, Center for Drug Evaluation and Research, U. S. Food and Drug Administration, Silver Spring, MD, United States; 5 CUBRC, Buffalo, NY, United States

**Keywords:** teriparatide, peptide drug, impurity, immunogenicity, computational immunology, HLA binding, T-cell assay, Treg epitope

## Abstract

Teriparatide is one of several generic peptides named in a recent Food and Drug Administration (FDA) guidance (FDA-2017-D-5767-0002), which outlines a potential strategy to inform immunogenicity risk assessment for synthetic generic peptides without requiring clinical studies. Specifically, the guidance states that for abbreviated new drug applications (ANDAs), once the sameness of the active pharmaceutical ingredient (API) between the generic product and the reference listed drug is established, developers can mitigate the residual risk of unwanted immunogenicity response by using *in silico* and *in vitro* tools to characterize differences in product- and process-related impurities between the reference and generic drug products. Regarding product-related impurities, a stated concern is that sequence modifications may create new T-cell epitopes capable of driving unwanted immune responses. Specifically, the guidance sets limits for the relative abundance of each impurity and requests that any new impurity above a certain concentration threshold be evaluated for potential T-cell-driven immunogenicity using orthogonal methods that assess both human leukocyte antigen (HLA) binding and the capacity to elicit a T-cell response. One such orthogonal immunogenicity risk assessment approach was applied to teriparatide (TPT) and several theoretical or observed product-related impurities in the case study described here. First, the immunogenic potential of TPT and selected impurities was assessed using three *in silico* tools: EpiMatrix, ClustiMer, and JanusMatrix. Second, an *in vitro* method was used to evaluate the binding affinity of TPT and the selected TPT impurities to different class II HLA-DRs *in vitro*. Third, a human peripheral blood mononuclear cell (PBMC) T-cell assay compared T-cell proliferation in response to individual impurities or the reference teriparatide drug product, Forteo^®^, *in vitro*. The orthogonal approaches identified multiple impurities as more immunogenic than TPT. In a novel finding, the *in silico* analysis revealed a potentially tolerogenic sequence in TPT, which correlated with lower-than-expected *de novo* immune responses to TPT *in vitro*. The analysis and methods described in this case study may help assess the relative risk of impurities and help identify those with the potential to increase the immunogenicity risk of a generic peptide.

## Graphical Abstract

**Figure:**
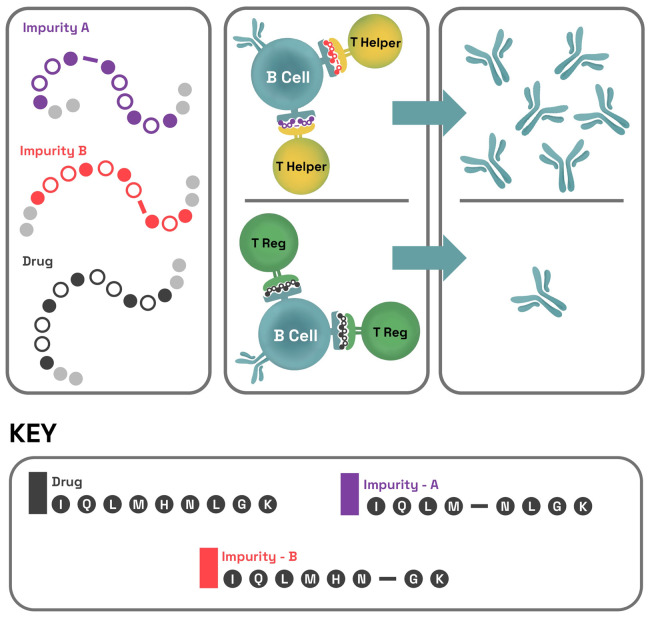

## Introduction

**Figure 1:**
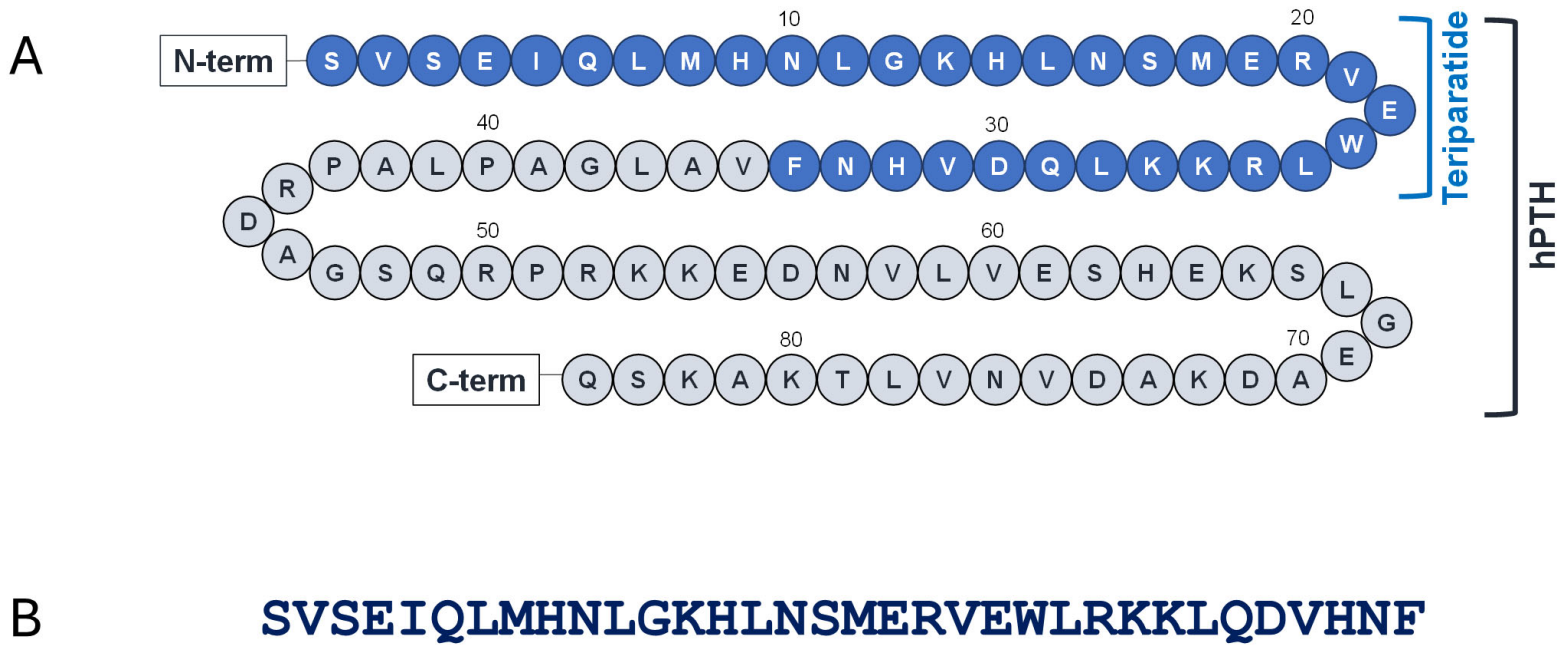
Overview of teriparatide sequence. **(A)** Sequence of human parathyroid hormone (hPTH). The N-terminal 34-amino acids (shaded blue) contain the biologically active region of the hormone and comprise the sequence of teriparatide. The C-terminus is involved in intracellular processing and secretion. **(B)** Teriparatide one-letter code sequence.

Teriparatide (TPT), sold under the brand name Forteo^®^, is a 34-amino acid peptide drug produced by recombinant DNA (rDNA) technology. Synthetic peptide versions of the drug have recently been approved for the generic drug market. TPT consists of the biologically active N-terminal 34 amino acids of human parathyroid hormone (PTH) (**Figure 1**) and is approved in the USA for the treatment of postmenopausal osteoporosis in patients at high risk of bone fracture. Since TPT’s sequence is identical to that of the N-terminus of PTH, the development of antibodies that cross-react with the endogenous hormone could potentially alter safety and efficacy. Clinically, however, the TPT drug product (Forteo^®^) is minimally immunogenic, with only 2.8% of treated patients developing antidrug antibodies (ADA) after 12 months of treatment (1).

In the 2021 synthetic peptide guidance (*FDA-2017-D-5767: ANDAs for certain highly purified synthetic peptide drug products that refer to listed drugs of rDNA origin*), the FDA recommends that generic peptide manufacturers submit information addressing the immunogenicity risk potential of peptide impurities that may be present in the final generic drug product. Specifically regarding product-related impurities or variants, the guidance states that manufacturers should “justify for each new specified peptide-related impurity … the ANDA applicant should provide justification for why such impurity does not affect the safety of the proposed generic synthetic peptide (including with respect to immunogenicity) and why it does not affect its effectiveness…. Such data should demonstrate for each new impurity that the impurity does not contain sequences that have an increased affinity for major histocompatibility complexes known as T-cell epitopes” (2). The recommended approach to ANDA immunogenicity requirements has been reviewed in several publications (3, 4).

*In silico* and *in vitro* assays for immunogenicity risk assessment, which are currently used to evaluate the immunogenicity of biologics, have recently been adapted for assessing the immunogenicity risk of generic peptide drugs and their impurities (3). For this case study, members of the US Food and Drug Administration Center for Drug Evaluation and Research, Office of Generic Drugs (FDA CDER OGD), provided a list of 34 synthetic TPT impurities for evaluation and comparison with Forteo^®^, the TPT reference listed drug product (RLD). These impurities were identified from internal studies and not from ANDA sponsor applications (**Supplementary Table S1**). Seven of the impurities were selected for further *in vitro* study. Selection was based on the following criteria: five “significant-risk” impurities, defined by a high EpiMatrix Score/low JanusMatrix Score (increased epitope content, reduced human homology, considered likely to be immunogenic), and two “low-risk” impurities defined by a reduced EpiMatrix Score/higher JanusMatrix Score (low immunogenicity was expected). The rationale for impurity selection also considered synthesis feasibility. Amino acid duplications, deletions, and side-chain modifications were prioritized, while truncations were not selected due to differences in peptide length and the absence of impurity-specific new epitope content.

The immunogenicity risk assessment of TPT and selected peptide-related impurities was performed using three orthogonal methods, as previously described (3). First, TPT and the selected impurities were assessed using well-established *in silico* tools (EpiMatrix, ClustiMer, and JanusMatrix) (3). Second, *in vitro* human leukocyte antigen (HLA) binding assays were conducted to evaluate differences in HLA binding affinity of TPT and its impurities across common HLA-DR allele supertypes. Third, T-cell responses elicited by Forteo^®^ and its impurities were compared *in vitro* using human peripheral blood mononuclear cell (PBMC) assays. In addition, a computational “what-if” study was performed with the “What-If-Machine” (WhIM) algorithm. WhIM is an iterative algorithm that starts with a baseline peptide sequence and generates lists of peptides with single modifications, representing theoretical impurities. The results for both sets of peptides (FDA-identified and WhIM-generated) are described in this case report.

**Figure 2:**
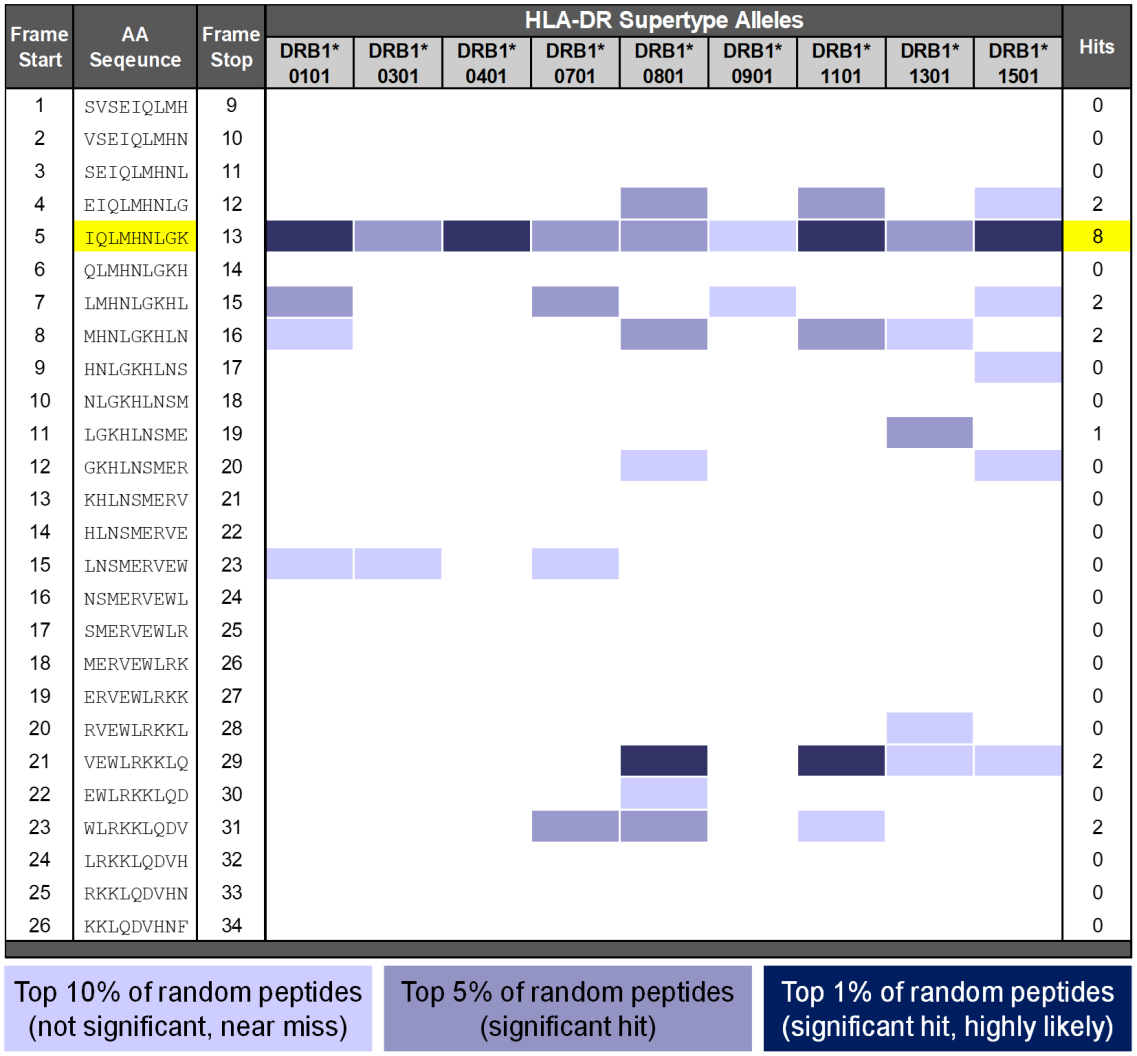
EpiMatrix analysis of teriparatide API. EpiMatrix detail report. The potential of a 9-mer frame to bind to a given HLA allele is indicated by a *Z*-score (scores omitted for simplicity); the strength of the score is indicated by the blue shading. All scores in the top 5% of the normal distribution are considered “Hits” (medium and dark blue shading). Scores in the top 10% are considered elevated but not significant (light blue shading). Frames containing four or more alleles scoring in the top 5% are highlighted in yellow. These frames have an increased likelihood of binding to a range of HLA alleles. The 9-mer epitope-bar (EpiBar) highlighted in yellow is a putative promiscuous epitope.

**Figure 3:**
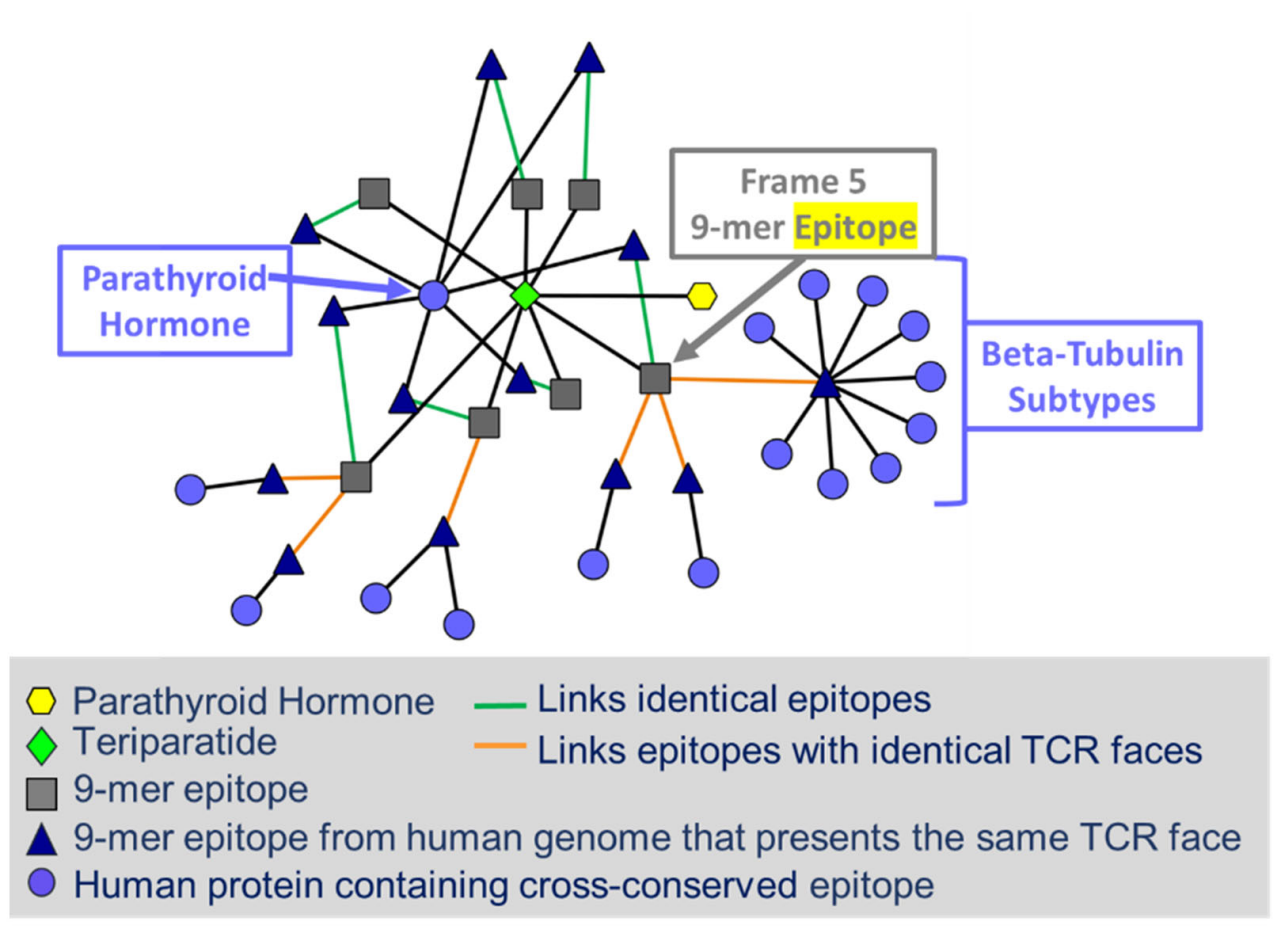
Cytoscape image of the JanusMatrix network for the teriparatide sequence. Given a peptide sequence (green diamond), EpiMatrix is used to identify 9-mer MHC ligands (grey squares). The JanusMatrix algorithm is subsequently used to search the human proteome reference database, considering the amino acid content of both the HLA-facing (binding) agretope and the TCR-facing epitope. Reference sequences with a compatible HLA binding agretope and exactly matching TCR contacts (epitope) of the input peptide and their source proteins are returned (blue circles and black triangles, respectively). When comparing peptide epitopes to the human proteome, JanusMatrix human homology scores above three (signifying that the human proteome contains more than three TCR-matched homologs of the input peptide or protein) are considered to be significant, indicating a greater-than-expected level of conservation between the TCR-facing features of the input peptide or protein and the TCR-facing features of proteins resident within the human genome. For a given EpiMatrix score, a high JanusMatrix score suggests a bias toward immune tolerance.

The *in silico* analysis of the parental TPT sequence using EpiMatrix (5) identified a promiscuous T-cell epitope in frame 5 (amino acids 5 to 13). This 9-mer amino sequence was predicted to bind multiple HLA-DR alleles (**Figure 2**). Interestingly, although high immunogenicity might be anticipated due to the presence of this potentially promiscuous T-cell epitope, assessment using the JanusMatrix tool—which compares the T cell receptor (TCR)-facing residues of T-cell epitopes with sequences derived from the human genome—revealed significant conservation of this sequence across multiple (non-PTH) proteins found in the human proteome (specifically, human β-tubulin, **Figure 3**) (3, 4). Prior research on cross-conserved epitopes similar to this one suggests that, rather than driving immunogenicity (4), the promiscuous T-cell epitope in frame 5 may be tolerated by the human immune system or actively tolerogenic.

Indeed, when comparing the TPT RLD, Forteo^®^, with nine TPT impurities using the naïve donor T-cell assay, we observed that several impurities in which the sequence of the promiscuous cross-conserved epitope was altered were more immunogenic *in vitro* than TPT. This finding suggests that impurities disrupting the tolerogenic epitope could contribute to immunogenicity in clinical use.

As demonstrated in this case study, each risk assessment method provided independent (orthogonal) perspectives on the potential immunogenicity of the individual TPT peptide-related impurities, and the results from the independent assays were largely aligned. Combining *in silico* analysis with *in vitro* studies may therefore help identify which impurities are likely to contribute to immunogenicity risk, guiding drug developers on which impurities should be controlled to reduce risk and alleviating concerns that might otherwise impede approval.

## Materials and methods

### Bioinformatics analysis screening of TPT and TPT impurities

The TPT active pharmaceutical ingredient (API) and FDA-approved impurities were evaluated using immunoinformatics analyses with the EpiMatrix and JanusMatrix algorithms (3, 6, 7). Several TPT impurities were selected for this study because they contained alterations to the promiscuous HLA-DR epitope in frame 5—such as amino acid insertions, deletions, or side-chain modifications—that reduced the epitope’s conservation with human proteome-derived sequences.

In some cases, impurity sequences contained unnatural or otherwise modified amino acid residues. While the HLA binding properties of peptides with unnatural amino acids cannot be directly estimated using standard *in silico* methods, a three-step approach was used to estimate the HLA binding properties of the modified sequence. Briefly, the modified position is substituted with all 20 naturally occurring amino acids, generating a range of possible scores to estimate the potential impact of the impurity modification on HLA binding. A wide range of scores for substitutions at a given position indicates that position may have a greater impact on predicted HLA binding, whereas a narrow range suggests that a modification at that position is unlikely to significantly impact the HLA binding affinity. Finally, a suitable natural amino acid substitution is selected for further analysis. This three-step approach for estimating HLA binding of sequences containing unnatural amino acids *in silico* has been described in greater detail previously (8).

**Figure 4:**
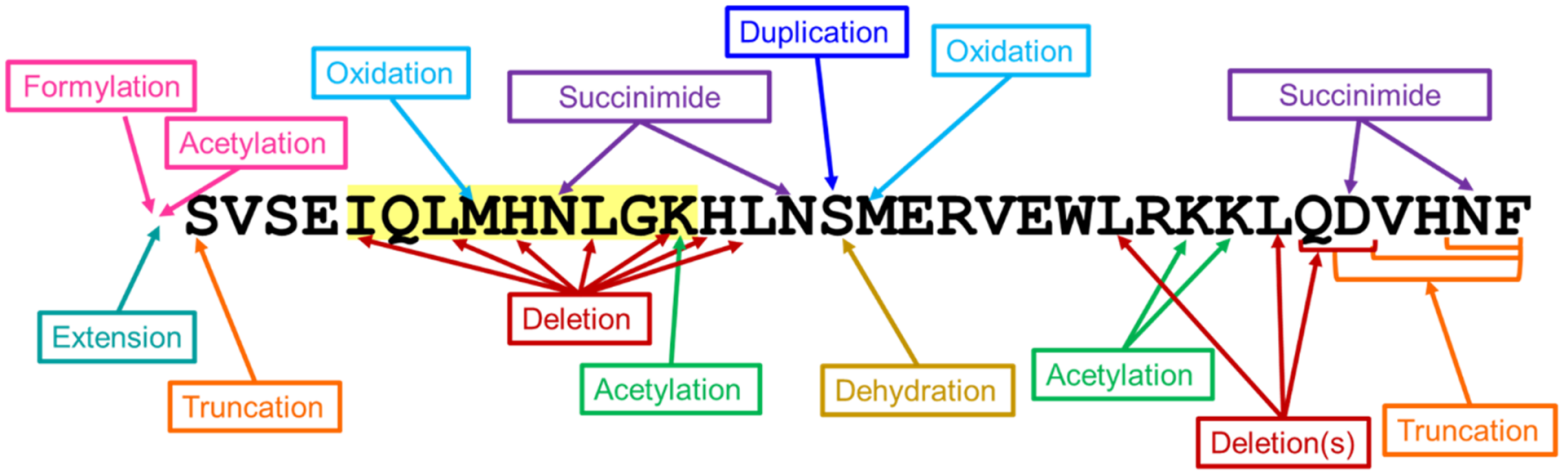
Impurity modifications on teriparatide sequence. Thirty-four teriparatide impurity peptides were analyzed for immunogenic potential *in silico*. The modifications to the teriparatide API sequence are shown above. The predicted tolerogenic epitope is highlighted in light yellow. Impurity modifications in this region, particularly in the TCR-facing residues of the frame 5 epitope, have the potential to reduce the predicted tolerance, potentially leading to a breach of tolerance and significant immune responses.

Having defined the approach to handling unnatural amino acids in selected impurities, EpiMatrix was used to identify T-cell epitopes in the sequences of the TPT API and 34 impurities (**Figure 4**). Each sequence was parsed into overlapping 9-mer frames and evaluated against a panel of nine class II supertype alleles (HLA-DRB1*0101, HLA-DRB1*0301, HLA-DRB1*0401, HLA-DRB1*0701, HLA-DRB1*0801, HLA-DRB1*0901, HLA-DRB1*1101, HLA-DRB1*1301, and HLA-DRB1*1501) to assess binding likelihood (3). These “supertype” alleles represent the binding preferences of HLA-DRB1 alleles for approximately 95% of the global population, and their selection has been described in detail elsewhere (7).

Next, the JanusMatrix algorithm was employed to determine whether the API and its impurities contained sequences that could be tolerated or tolerogenic. JanusMatrix identifies putative T-cell epitopes in protein and peptide sequences that, while not necessarily identical in sequence to known human T-cell epitopes, are conserved at their TCR-facing residues and retain the same HLA binding restriction. Many such epitopes have been shown to be tolerated or even tolerogenic *in vitro* and *in vivo* when extensive cross-conservation with the human proteome is observed (6, 9). As demonstrated for highly cross-conserved and tolerogenic T-cell epitopes, modification of the TCR-facing residues typically reduces their tolerogenic properties (10, 11). Similarly, a reduction in the cross-conservation of the frame 5 promiscuous and cross-conserved epitope in the API might lead to increased immunogenicity *in vitro* and *in vivo*, as discussed in greater detail below.

Finally, the impurity sequences were assessed for their potential to increase the immunogenicity of the drug product due to the presence of new epitopes predicted in the impurity but not in the API (3). Specifically, the analysis prioritized amino acid modifications likely to introduce new HLA ligands and/or new TCR-facing contours compared to the unmodified amino acid sequence. In the context of immunogenicity risk assessment, both types of these newly created epitopes (new ligands and new TCR-facing residues) can be considered “new epitopes” to the host immune system (different from those in the API).

This emphasis on the creation of new epitopes that are not conserved with the API relates to the additive effect of effector T-cell responses to these epitopes. Immune responses to new epitopes could induce or enhance immune responses targeting the active drug ingredient through a process known as “epitope spreading” (12–15). In contrast, T-cell epitopes that are identical in both the unmodified product and the amino acid sequences of product impurities (common epitopes) may engage and activate cognate T cells but are considered unlikely to induce *new* immune responses and are not expected to contribute to an *increase* in immunogenicity.

In addition to prioritizing new epitopes, *in silico* assessments emphasize the identification of promiscuous binding sequences that are likely to bind to multiple HLA-DR alleles. Impurities containing modifications in these promiscuous sequences are likely to introduce novel epitopes capable of inducing CD4^+^ T-cell responses across multiple alleles (16, 17); for this reason, such impurities are prioritized for evaluation both *in silico* and *in vitro*.

### Generation of peptide impurities using the What-If-Machine algorithm

A new algorithm was developed for this case study that can be used to explore multiple potential impurities that may impact the immunogenicity of a generic peptide. This algorithm, called the What-If-Machine (WhIM), can be used to create a ranked list of potential impurities and prospectively identify impurities with sequences that would have high (or low) immunogenic risk potential (18). WhIM is an iterative algorithm that produces lists of theoretical impurity peptides, each containing a single modification from a baseline sequence (generally the API sequence). WhIM records possible sequence-related impurities created through known failures in the synthesis process (19). WhIM mimics the solid-phase peptide synthesis process, in which each addition of an amino acid from the C-terminus to the N-terminus is considered a cycle. At each cycle, WhIM documents theoretical impurities based on possible single modifications to the baseline sequence. These modifications include amino acid deletions, insertions, duplications, and side-chain modifications. Ultimately, the WhIM algorithm generates a list of theoretical impurities for a given API and then assesses each for its potential immunogenicity using EpiMatrix, ClustiMer, and JanusMatrix, and creates scores for each of the modified peptides.

**Figure 5:**
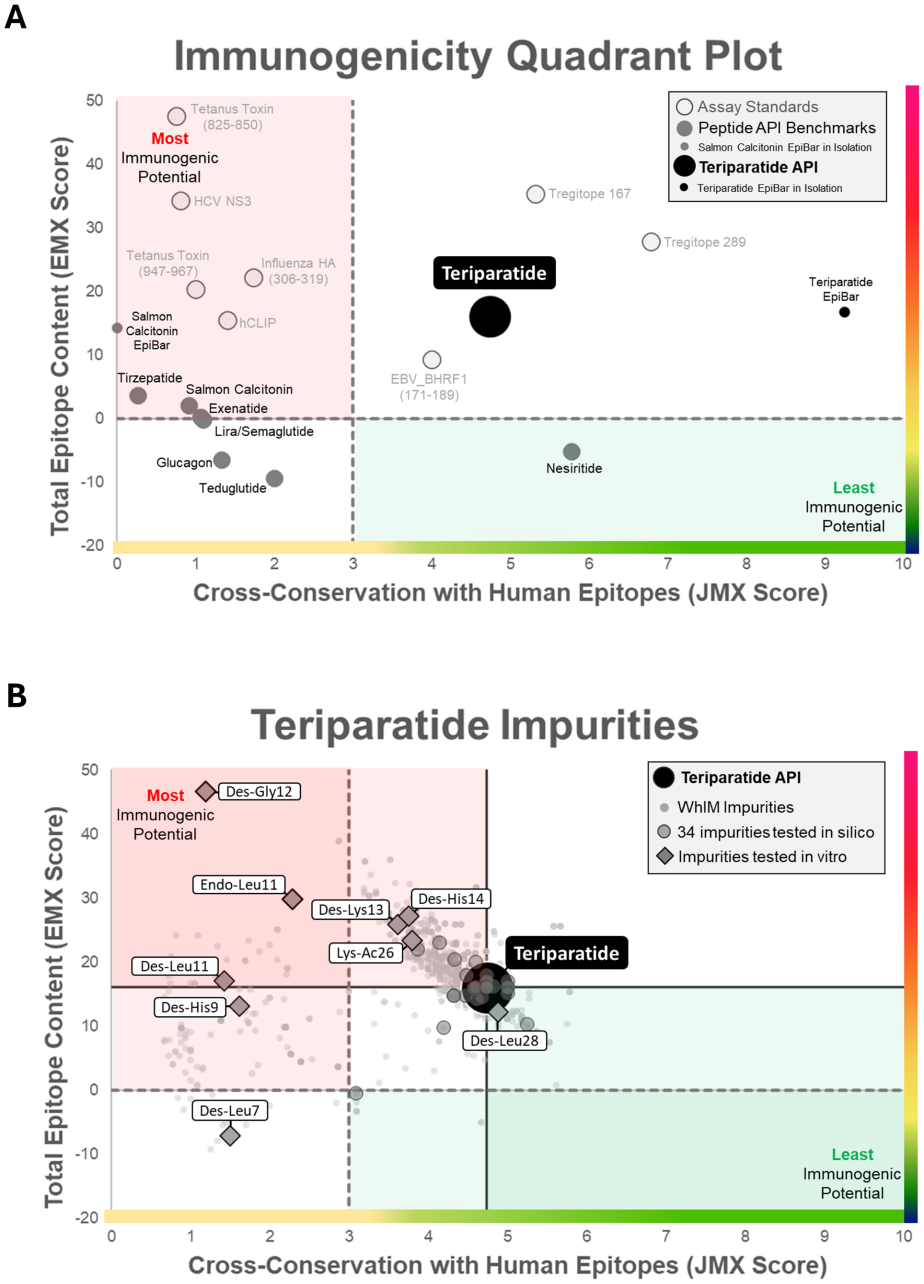
Immunogenicity quadrant plot. **(A)** Immunogenicity quadrant analysis categorizes peptides and impurities by immunogenicity risk. EpiMatrix (EMX) and JanusMatrix Human Homology (JMX) scores are plotted for each peptide and impurity. The graph is divided into four quadrants based on total T-cell epitope content and cross-conservation with human epitopes (dashed lines). Peptide API benchmarks are shown in grey circles to provide an *in silico* comparison with the TPT API (black circle), which is the focus of this case study. Peptides that fall into the top-left “epitope dense, less common in human proteins” quadrant (red shading) are likely to induce an immune response, while peptides and impurities that fall in the bottom-right “epitope sparse, more common in human proteins’ quadrant (green shading) are considered lower risk for inducing an immune response. **(B)** Teriparatide impurities. Here, teriparatide-related impurities are plotted on the immunogenicity quadrant plot and compared to both the standard quadrant thresholds described in **(A)** (dashed lines) and to the teriparatide API (solid lines indicate the “relative assessment” compared to the API). Scores for the WhIM-generated theoretical impurities are shown as small gray circles. The 34 teriparatide impurities analyzed *in silico* are shown as medium gray circles. Impurities selected for the *in vitro* IVIP T-cell assay study are depicted with diamond shapes. Impurities that increase overall epitope content and decrease humanness relative to the API (top left quadrants) are considered to have the highest immunogenicity risk potential.

Thousands of theoretical synthetic peptide impurities of TPT, resulting from sequence changes (insertions, deletions, duplications, and others), were designed using the tool. Following the generation of this extensive list of theoretical peptide-related impurities by WhIM, existing algorithms (EpiMatrix and JanusMatrix) were used to rank each member of the list for its potential to generate immune responses in global patient populations. These algorithms are described in greater detail in the **Supplementary Material** and in De Groot et al. (3) and Mattei et al. (5). The results of the WhIM analysis can be plotted on a quadrant plot showing their risk potential relative to defined EpiMatrix and JanusMatrix thresholds (and to the “observed” impurities tested in this study) (see **Figure 5**).

### Selecting peptides for *in vitro* assays

Following the *in silico* analysis of the 34 observed impurities (from the FDA) and thousands of WhIM-identified potential impurities, nine impurities were selected for further testing. These included two impurities selected to represent low-risk impurities (for impurity-induced immunogenicity) and five selected to represent high-risk impurities (based on their estimated immunogenicity) by *in silico* analysis. In addition, two high-scoring theoretical impurities designed by the WhIM were selected for evaluation *in vitro*.

As class II HLA binding peptides are usually 12–25-amino acid long, the peptides produced for *in vitro* assays were designed to capture the modified region and center any predicted binding epitopes in the middle of the peptide to be tested, while remaining consistent with standard class II peptide length limits. In general, peptides used in HLA-DR binding assays are shortened to improve the potential for the assay to capture the effect of the centered epitope, whereas peptides evaluated in T-cell assays are tested as full-length peptides (see **Table 1**) (20).

**Table 1:** Teriparatide impurities selected for *in vitro* assays.

Peptide name	Peptide sequence^a^	EMX hits	EMX score	JMX score
TERIPARATIDE	SVSEIQLMHNLGKHLNSMERVEWLRKKLQDVHNF (N-term: SVSEIQLMHNLGKHLNSM-NH2; C-term: MERVEWLRKKLQDVHNF-OH)	**19**	**16.03**	**4.74**
Observed impurities of teriparatide				
LYS-AC26_TERIPARATIDE	SVSEIQLMHNLGKHLNSMERVEWLR(K-Ac)KLQDVHNF	24	23.44	3.79
DES-LEU7_TERIPARATIDE	SVSEIQ–MHNLGKHLNSMERVEWLRKKLQDVHNF	8	-7.10	1.5
DES-HIS9_TERIPARATIDE	SVSEIQLM–NLGKHLNSMERVEWLRKKLQDVHNF	18	13.07	1.61
DES-LEU11_TERIPARATIDE	SVSEIQLMHN–GKHLNSMERVEWLRKKLQDVHNF	19	17.02	1.42
DES-LYS13_TERIPARATIDE	SVSEIQLMHNLG–HLNSMERVEWLRKKLQDVHNF	23	25.85	3.61
DES-HIS14_TERIPARATIDE	SVSEIQLMHNLGK–LNSMERVEWLRKKLQDVHNF	24	27.16	3.75
DES-LEU28_TERIPARATIDE	SVSEIQLMHNLGKHLNSMERVEWLRKK–QDVHNF	17	12.23	4.88
Theoretical impurities predicted by WhIM				
DES-GLY12_TERIPARATIDE	SVSEIQLMHNL–KHLNSMERVEWLRKKLQDVHNF	31	46.63	1.19
ENDO-LEU11_TERIPARATIDE	SVSEIQLMHNLLGKHLNSMERVEWLRKKLQDVHNF	24	29.72	2.29

This table provides details on the peptides tested in *in vitro* assays. Full-length peptides were synthesized for the *ex vivo* IVIP (T-cell) assay. EpiMatrix (EMX) hits represent the number of predicted HLA ligands in the full-length sequence. EpiMatrix (EMX) score indicates the score for the full-length sequence. JanusMatrix (JMX) Homology scores indicate the average depth of epitope cross-conservation with the human proteome; for human-derived peptides, JMX scores above 3.00 are considered to have an elevated potential for homology-induced tolerance. Red font highlights the impurity: a red hyphen (-) indicates an amino acid deletion at that position.

a Underlined amino acids indicate the shorter peptides synthesized for assessment in the class II HLA binding assay.The bold values establish the 'baseline" EMX and JMX values for the teriparatide API.

### TPT and impurity peptides

Impurity peptides used in the *in vitro* studies were synthesized by 21st Century Biochemicals (Marlborough, MA, USA). Molecular weight was verified by mass spectrometry, and all peptides were determined to be greater than 95% pure by HPLC. Forteo^®^ (RLD) drug product was purchased from Pharmaceutical Buyers Inc. (New Hyde Park, NY, USA).

### Class II HLA binding assays

Class II HLA binding assays used in these studies measure the binding affinity of a target peptide to HLA-DRB1*0101, HLA-DRB1*0301, HLA-DRB1*0401, HLA-DRB1*0701, HLA-DRB1*0901, HLA-DRB1*1101, HLA-DRB1*1301, and HLA-DRB1*1501. The HLA binding assay was originally described by Steere et al. (21) and adapted by EpiVax (22, 23). Briefly, unlabeled test peptides were incubated overnight to equilibrium with a soluble HLA-DR molecule (Benaroya Research Institute, Seattle, WA, USA) and a biotinylated, allele-specific competitor peptide of known binding affinity. The binding reaction was then neutralized with a buffered pH change, and the peptide-HLA complexes were transferred to a 96-well plate coated with a pan-HLA-DR antibody, clone L243 (BioLegend, San Diego, CA, United States), and incubated overnight. The following day, plates were resolved with the addition of europium-labeled streptavidin (Perkin-Elmer, Waltham, MA, USA). An indirect measure of binding was determined by time-resolved fluorescence. Each peptide was evaluated in triplicate over a range of seven concentrations. The percent inhibition values for each experimental peptide across this concentration range were used to calculate an IC_50_, the concentration at which the peptide inhibits 50% of the labeled competitor peptide.

### Human peripheral blood mononuclear cells

PBMCs used in these assays were freshly isolated from leukocyte reduction filters purchased from the Rhode Island Blood Center (RIBC) in Providence, RI. High-resolution (four-digit) class II HLA haplotyping of donors was performed at the Transplant Immunology Laboratory at Hartford Hospital (Hartford, CT, USA) using the sequence-specific oligonucleotide method. Donor HLA-DRB1 types, age, and sex are provided in **Supplementary Data** (see **Supplementary Figure S1**). All Donor PBMC samples had greater than 85% viability, postisolation.

### *In vitro* immunogenicity protocol

To measure the naïve CD4^+^ T-cell response to TPT and impurities, freshly isolated PBMCs were plated at a density of 2.5 × 10^5^ cells per well in 96-well U-bottom cell plates in RPMI-1640 cell culture media supplemented with IL-2 (10 ng/mL) and IL-7 (20 ng/mL) (Gibco, Grand Island, NY, United States). For each test article and control, nine replicate wells of 2.5 × 10^5^ cells (2.25 × 10^6^ cells total) were plated. Cells were incubated with Forteo^®^ (the approved TPT RLD product) or individual impurities at 20 µg/mL for 14 days at 37°C/5% CO_2_. Micromolar (µM) concentrations for peptides evaluated in the *in vitro* immunogenicity protocol (IVIP) assay are provided in **Table 2**, below (data for impurities evaluated at 0.2 µg/mL is provided in **Supplementary Figure S4**). Media exchanges, including cytokine support (IL-2 and IL-7) but no antigen, were performed on days 4, 7, and 11. The *in vitro* concentration of 20 µg/mL was chosen based on a dose-optimization study performed on Forteo^®^ to determine the maximum dose at which a T-cell response could be observed with no toxicity to the cells.

**Table 2:** Peptide concentrations (µM) evaluated in the IVIP assay.

Peptide	20 µg/mL concentration	0.2 µg/mL concentration
Teriparatide (in Forteo^®^)	4.86 µM	0.05 µM
Impurity LysAC26	4.81 µM	0.05 µM
Impurity Des-Leu7	4.99 µM	0.05 µM
Impurity Des-His9	5.02 µM	0.05 µM
Impurity Des-Leu11	4.99 µM	0.05 µM
Impurity Des-Lys13	5.01 µM	0.05 µM
Impurity Des-His14	5.02 µM	0.05 µM
Impurity Des-Leu28	4.99 µM	0.05 µM
WhIM Impurity Des-Gly12	4.93 µM	0.05 µM
WhIM Impurity Endo-Leu11	4.73 µM	0.05 µM

Each set of donor PBMC was plated with the test peptides as well as the following sets of controls: positive control: keyhole limpet hemocyanin (KLH; Thermo Fisher, Waltham, MA, United States) and the antigenic memory peptide pool Cytomegalovirus, Epstein–Barr virus, and influenza (CEFT; ImmunoSpot, Cleveland, OH, United States); negative control: human serum albumin (HSA; Sigma-Aldrich, Saint Louis, MO, United States); and Functional Control: Phytohemagglutinin (PHA; Thermo Fisher), a well-known T-cell mitogen. Each test article and control peptide was tested in nine replicate wells. Only donor PBMC that generated a positive response to KLH, CEFT, and PHA, and a negative response to HSA were included in the study.

To compare the impact of the Forteo^®^ formulation on cell viability and immunogenicity, impurity peptides evaluated in these assays were reconstituted in either supplemented RPMI-1640 cell culture media or a diluent formulated to replicate the Forteo^®^ product formula (1), which contained: 0.41 mg/mL glacial acetic acid, 0.1 mg/mL sodium acetate anhydrous, 45.4 mg/mL mannitol, 3.0 mg/mL metacresol, and pH 4.0, as specified in the Forteo^®^ package insert.

Please see the discussion for an update on this approach that resulted from this and other studies conducted in this EpiVax-FDA collaboration.

### Fluorospot assay

Following the 14-day incubation, cells were harvested and plated in triplicate on a precoated antihuman interferon gamma (IFN-γ) Fluorospot plate (Mabtech, Cincinnati, OH, United States) at a concentration of 1.0 × 10^5^ cells per well in the presence of the appropriate test article in RPMI-1640 culture media. Fluorospot plates were incubated for 48 h at 37°C/5% CO_2_. After 48 h, Fluorospot plates were developed according to the manufacturer’s instructions. Fluorospot plate counting was performed by unbiased experts at ZellNet Consulting Inc. (Fort Lee, NJ, USA) as previously described by Roberts et al. (24). A response was considered positive if (1) the number of spots was at least twice the background (SI ≥ 2), and (2) there were greater than 50 spot-forming cells (SPC) per 1,000,000 PBMCs.

### Tetanus toxoid bystander suppression assay

To evaluate the regulatory potential of predicted regulatory T-cell (Treg) epitope peptides identified in TPT, a previously published tetanus toxoid bystander suppression assay (25) was adapted to determine if predicted Treg epitopes could suppress the memory CD4^+^ T-cell response to the common tetanus toxoid antigen. Cryopreserved PBMCs were thawed, the viability was measured postthaw using AO/PI viability dye on a Nexelcom Cellometer (Nexelcom, Lawrence, MA, United States), and then labeled with carboxyfluorescein succinimidyl ester (CFSE, eBioscience, San Diego, CA, United States). The CFSE-labeled cells were plated in 96-well U-bottom culture plates at a concentration of 3.0 × 10^5^ cells per well in RPMI-1640 cell culture medium supplemented with 2 nM l-glutamine, 50 µg/mL gentamicin (Life Technologies, Beverly, MA, United States), 10% human AB serum (Sigma), and MEM nonessential amino acids and 55 µM β-mercaptoethanol (Gibco), known as complete RPMI-1640 (cRPMI-1640) and rested overnight at 37°C/5% CO_2_. The putative regulatory TPT peptide was solubilized in dimethyl sulfoxide (DMSO) and further diluted to assay concentrations in cRPMI-1640 culture medium. At day 1, cells were stimulated with tetanus toxoid (TT; Astarte Biologics, Redmond, WA, United States) at 0.5 µg/mL alone for positive control of the experiment or in combination with putative peptide TPT Treg epitope, in a series of concentrations at 0, 10, 20, and 40 µg/mL and incubated for 7 days at 37°C/5% CO_2_. After 7 days, cells were stained for the expression of cell surface markers and analyzed by flow cytometry (see below). Wells treated with TT alone and TT^+^ Treg epitope-treated cells were compared after 7 days. TPT was compared with two well-characterized Treg epitopes (Tregitope 289 and FV621) that had been previously identified and extensively validated (26–30). Negative control peptides have been included in previous assays and compared with the positive controls used in this study (see, for example (31)).

### Flow cytometry

Cells were washed with 1× PBS and stained with live/dead viability stain (Life Technologies, Beverly, MA, United States) according to standard procedures. Cells were then washed with 1× FACs buffer (1× PBS+5% FBS), and surface-stained for CD3 (clone OKT3), CD4 (clone OKT4), and CD25 (clone BC96) (BioLegend) in 1× PBS+5% FBS at 4°C for 30 min. Stained cells were washed twice with FACS buffer and acquired on an Attune NxT Acoustic Cytometer with three laser capacity (violet: 405 nm; blue: 488 nm; and red: 637 nm; Life Technologies) and analyzed for proliferation (dilution of CFSE) and surface antigen expression using FlowJo Software (Treestar Inc., Mesa, AZ, United States).

## Results

### Results of an *in silico* study of TPT impurities

A detailed *in silico* analysis of the TPT API sequence is presented in **Figure 2**. TPT contains 19 predicted HLA-DRB1 ligands, or EpiMatrix “hits”, which is more than one would expect to find in a random peptide of equivalent length, and consequently, TPT falls in the elevated range on the immunogenicity scale (greater than 10) based on total predicted T-cell epitope content. Eight of the 19 EpiMatrix hits were concentrated within a promiscuous T-cell epitope (highlighted in yellow), located at frame 5. Due to the extensive HLA binding potential in frame 5, the overall score of the sequence was high, and the promiscuous binding region in the frame 5 epitope was predicted to be a relatively high-affinity HLA ligand. Evaluation for cross-conservation with the human genome using the JanusMatrix algorithm, however, revealed extensive human genome epitope homology at the TCR-facing residues of this peptide (see below). Additional EpiMatrix hits that may be immunogenic are in frames 4, 7, 8, 11, 21, and 23. While these additional hits may be T-cell epitopes, their individual effects are expected to be more HLA restricted (as they are not within a promiscuous T-cell epitope).

The JanusMatrix algorithm was employed to screen the putative T-cell epitopes identified within TPT against the human proteome (**Figure 3**). The TPT sequence shares a high number of TCR-facing contours with peptides derived from self, resulting in a JanusMatrix human homology score of 4.74 (a high score, indicating that the peptide is likely to be tolerogenic). Specifically, the 9-mer peptide in frame 5 shares a TCR-facing pattern with epitopes derived from 12 human proteins, including peptides from parathyroid hormone (as expected) and numerous subtypes of β-tubulin (a ubiquitous structural protein in human cells), integrator complex, and insulin receptor-related protein. The HLA ligand in frame 11 was related by JanusMatrix to three human proteins at its TCR face, including peptides derived from from parathyroid hormone and annexin. The HLA ligands in frame 23 could be related to three human proteins at its TCR face, including peptides derived from parathyroid hormone, WASH complex, and desmocollin-1. The HLA ligands in frames 4, 7, 8, and 21 share some homology with parathyroid hormone at the TCR face.

As a result of the extensive homology with human genome epitopes uncovered in this analysis, it was postulated that the TPT epitope (frame 5) sequence-specific T cells may have either been deleted during thymic development, rendered anergic, or adopted a Treg phenotype. Consequently, changes to this 9-mer region, particularly those that abrogate homology with other peptides in the human proteome, might be detrimental to homology-induced tolerance and lead to increased immunogenicity risk (31).

### Selection of impurities for *in vitro* studies

The 34 observed impurities evaluated *in silico* included impurities resulting from amino acid deletions, amino acid duplications, side-chain modifications (including oxidation, succinimide formation, dehydration, acetylation), N- and C-terminal truncations, and N-terminal extension and modification (**Figure 4**). These peptide-related impurity sequences were specifically evaluated for new immunogenic (nonhuman-like) epitope content relative to the TPT API sequence (**Supplementary Table S1****).**

The EpiMatrix immunogenicity scores of the submitted impurity sequences (**Supplementary Table S1**) varied from low- to high-risk potential (higher EpiMatrix scores denote higher T-cell epitope content). Additionally, the JanusMatrix (human homology) analysis of the impurity sequences resulted in scores ranging from 1.42 (potentially immunogenic) to 5.25 (potentially tolerated or tolerogenic). Some impurity modifications, therefore, resulted in sequences with higher EpiMatrix scores and reduced JanusMatrix scores, and consequently, potentially higher immunogenicity risk.

Relative to the TPT API, we hypothesized that some modifications had the potential to reduce tolerogenic epitope content, which could drive an impurity-induced immune response. In these cases, the peptide impurities assessed had (1) equivalent, (2) elevated epitope content, and/or (3) reduced human homology relative to the TPT API. Impurities with elevated epitope content (higher EpiMatrix scores) and low human homology (low JanusMatrix scores) were identified as the most likely to be immunogenic.

**Figure 5A** shows the immunogenicity quadrant plot and relevant benchmarks. TPT falls into the top right quadrant, indicating that, while it contains a high amount of predicted epitope content, its epitopes were highly conserved with epitopes derived from the human proteome. Peptides residing in this quadrant include known tolerogenic control peptides, suggesting that the API may also be tolerogenic. In contrast, peptides known to be immunogenic can be observed in the top left quadrant, which carries the highest immunogenicity risk. Human-derived peptides with clinically low immunogenicity can sometimes fall in the left quadrants, indicating low JanusMatrix human homology scores due to their lack of cross-conservation with epitopes derived from additional autologous proteins cataloged in the JanusMatrix (UniProt, Geneva, Switzerland) database of human proteins, beyond that from which the database was derived (see glucagon, lira/semaglutide). **Figure 5B** shows the results of the *in silico* analysis of theoretical TPT impurities identified with the WhIM algorithm (small gray circles), as well as the potential immunogenicity of the 34 observed TPT impurity sequences (medium gray circles), as defined by their EpiMatrix score (*y*-axis) and their JanusMatrix score (*x*-axis), relative to the TPT API (large black circle). Those impurities that maintain the tolerogenic sequence in frame 5 are co-located with the parent peptide on this plot and are also considered lower risk for immunogenicity. Note that other TPT impurity sequences that have increased or equivalent T-cell epitope content and reduced human homology compared to the TPT API move up and left on the plot into the higher-risk quadrants (upper left). Several of these impurities (diamond-shaped on the quadrant plot) were found to have an enhanced immunogenicity risk potential when compared to the API peptide.

### Results of class II HLA binding assays

Following the *in silico* analysis, epitope binding predictions by EpiMatrix were evaluated *in vitro* by measuring the binding affinity of each impurity peptide relative to the corresponding N- or C-terminal portion of the TPT API, *in vitro*. The class II HLA-DRB1 binding assay was adapted from an assay originally described by Steere et al. (21) that provides an indirect measurement of peptide-HLA affinity. Class II HLA binding peptides typically range in length from 12–20 amino acids; however, the core HLA binding region is nine amino acids. The class II HLA binding groove is open-ended, and the additional flanking residues on either end of the core binding motif stabilize the peptide: HLA binding interaction. Targeted binding motifs were properly centered within the shorter length peptides synthesized for these binding studies (**Table 3**).

**Table 3:** Sequence of teriparatide and selected impurities evaluated in the HLA class II binding assay.

Impurity terminal location in peptide	Peptide name	HLA binding assay peptide sequence
N-terminal peptides	API: teriparatide	SVSEIQLMHNLGKHLNSM
	IMP: DES-LEU7_TPT (1–17)	SVSEIQ–MHNLGKHLNSM
	IMP: DES-HIS9_TPT (1–18)	SVSEIQLM–NLGKHLNSME
	IMP: DES-LEU11_TPT (1–18)	SVSEIQLMHN–GKHLNSME
	IMP: DES-LYS13_TPT (1–21)	SVSEIQLMHNLG–HLNSMERVE
	IMP: DES-HIS14_TPT (1–21)	SVSEIQLMHNLGK–LNSMERVE
	WhIM: DES-GLY12_TPT (1–21)	SVSEIQLMHNL–KHLNSMERVE
	WhIM: ENDO-LEU11_TPT (1–21)	SVSEIQLMHNLLKHLNSMERVE
C-terminal peptides	API: teriparatide	MERVEWLRKKLQDVHNF
	IMP: LYS-AC26_TPT (18–34)	MERVEWLR(K-Ac)KLQDVHNF
	IMP: DES-LEU28_TPT (18–33)	MERVEWLRKK–QDVHNF

Short peptides were designed to center the predicted epitope content for the API, the seven observed impurities, and two WhIM-generated impurities for evaluation in HLA binding. Red font highlights the impurity: red hyphen (-) indicates a deletion at that position. See **Table 1** to compare these peptides with their source impurities.

**Figure 6:**
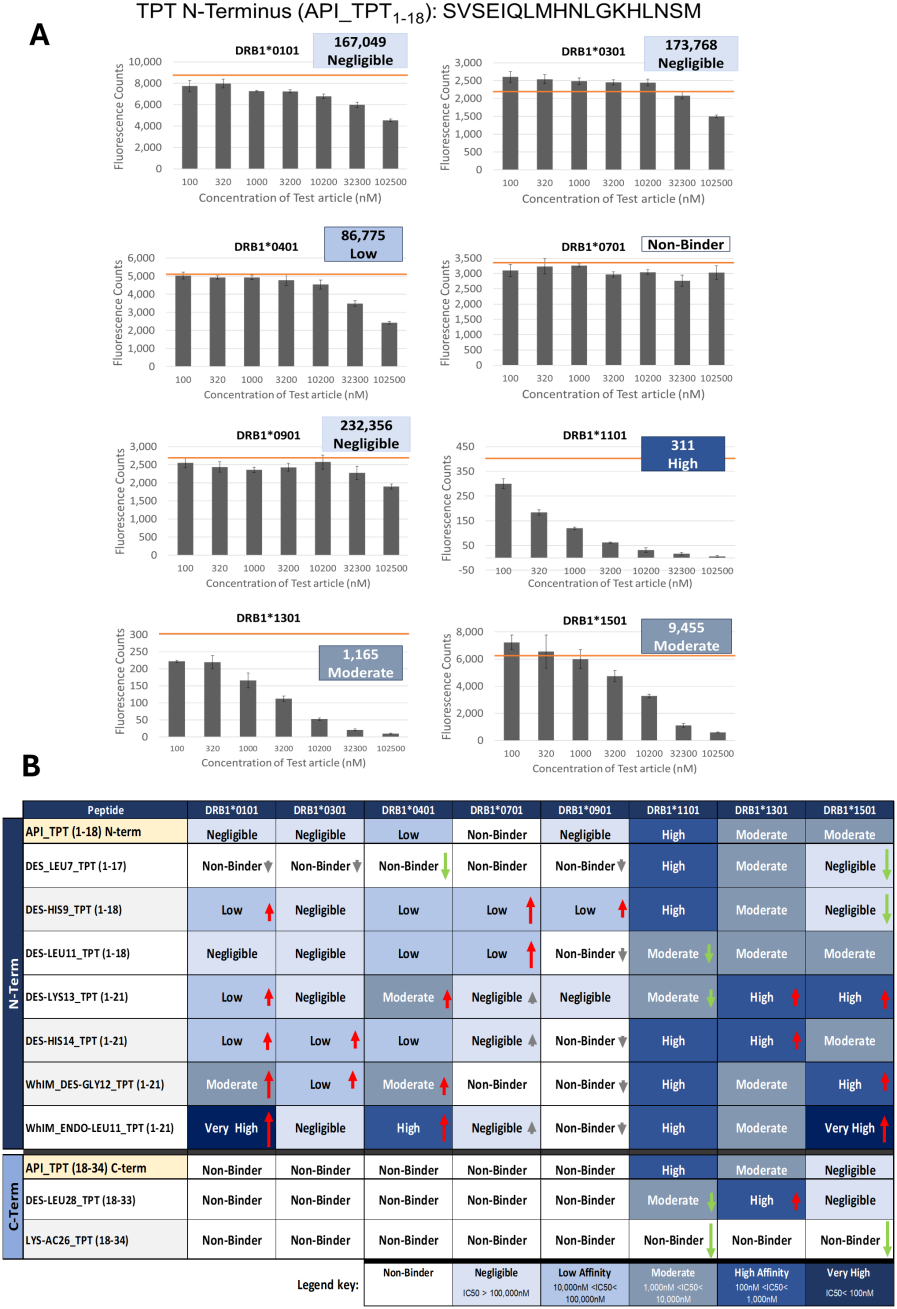
Results of the class II HLA binding assay. **(A)** Results of the HLA binding assay are shown for the N-terminal API TPT (1–18) peptide. The orange bar on each graph denotes the maximum fluorescence value (no inhibition). Each allele was run as a separate assay with its own specific labeled tracer peptide; consequently, the scales representing fluorescent counts differ between alleles. **(B)** High-level summary of the results of *in vitro* class II HLA binding assays. TPT was divided into the N- and C-terminal halves to allow direct comparison of the peptides derived from impurities. *Top half of the table:* results for the N-terminal TPT API peptide and its N-terminal impurities. *Bottom half of the table:* the C-terminal peptide and its two impurities. In addition to the observed impurities, two high-scoring theoretical impurities identified by the WhIM were selected for assessment in these *in vitro* assays (sequences are listed in **Table 2**). *HLA binding affinity:* blue-shaded boxes indicate the binding affinity as determined by the IC_50_ value for each peptide/HLA-DRB1 assessment. Affinity categories are defined in the legend key as follows: very high affinity corresponds to IC_50_ values less than 100 nM; high affinity ranges from 100 to less than 1,000 nM; moderate affinity ranges from 1,000 to less than 10,000 nM; low affinity ranges from 10,000 to less than 100,000 nM; and negligible affinity corresponds to IC_50_ values equal to or greater than 100,000 nM. Nonbinders show no dose–response even at 100,000 nM. *Relative binding comparisons:* Red arrows (↑) represent an increase in binding affinity relative to the comparator API peptide, and Green arrows (↑) represent a decrease in binding affinity. Gray arrows (↑↓) indicate a slight change in affinity that is not significantly different from the comparator API peptide (shown in the yellow shaded box). The length of arrows indicates the magnitude of the change.

The HLA binding results demonstrate that the N-terminus of TPT (which contains a promiscuous HLA binding motif in frame 5) bound to seven of the eight class II Supertype Alleles evaluated in these assays with negligible to high affinity (**Figure 6A**). These results support the *in silico* EpiMatrix assessment of the N-terminal promiscuous T-cell epitope (frame 5), as the assessment suggested promiscuous binding behavior. The HLA-DR promiscuity of this peptide may contribute to its activity as a Treg epitope (because the peptide may be tolerogenic for most subjects). These assays also independently confirmed that the C-terminus of TPT (which was assessed by EpiMatrix as having very few T-cell epitopes) bound to only three of the eight class II alleles tested, supporting the hypothesis that any immunogenic risk these impurities may pose would be HLA-restricted (**Figure 6B**).

To determine if the impurity modifications impacted class II HLA-DRB1 binding, impurity peptides were evaluated alongside their respective TPT API peptide (N-term or C-term) in HLAs. The resulting data is summarized in **Figure 6B** below. (Detailed results for each peptide-allele assessment are available in **Supplementary Figure S2**). Overall, most impurities showed equivalent or increased affinity as compared to the corresponding region of the API. This was consistent with the EpiMatrix predictions of equivalent or increased epitope content in these impurities relative to the API. By contrast, impurity Des-Leu7 was predicted by EpiMatrix to significantly reduce overall epitope content and likewise showed decreased or equivalent binding affinity compared to the corresponding region of the API.

### Results of IVIP T-cell assays

As previously mentioned, the low incidence of observed clinical immunogenicity associated with TPT may be attributed to (a) TPT being derived from the N-terminus of human PTH (an endogenous peptide) and/or (b) due to the presence of a potential Treg epitope in the N-terminus that is cross-conserved with β-tubulin and other self-proteins. Of significant concern is whether or not impurities that make the peptide appear more foreign (by changing the tolerogenic epitope) might impact the immunogenicity of the drug product. To assess the immunogenicity of the impurities relative to the RLD product, Forteo^®^, PBMCs from healthy donors were incubated with individual impurity peptides or the Forteo^®^ drug product at an equal concentration to the API.

Data collected during a previous analysis of salmon calcitonin (24) and innate immunogenicity studies conducted by Holley et al. (32) indicated that excipients within the formulation could impact cell health and viability in culture. Thus, synthetic peptide impurities were reconstituted in either cell culture media, or a buffer made in our laboratory that was made to mimic the Forteo^®^ product buffer (0.41 mg/mL of glacial acetic acid, 0.1 mg/mL of sodium acetate anhydrous, 45.4 mg/mL of mannitol, 3.0 mg/mL of metacresol, pH 4.0 as specified in the Forteo^®^ package insert) (1).

**Figure 7:**
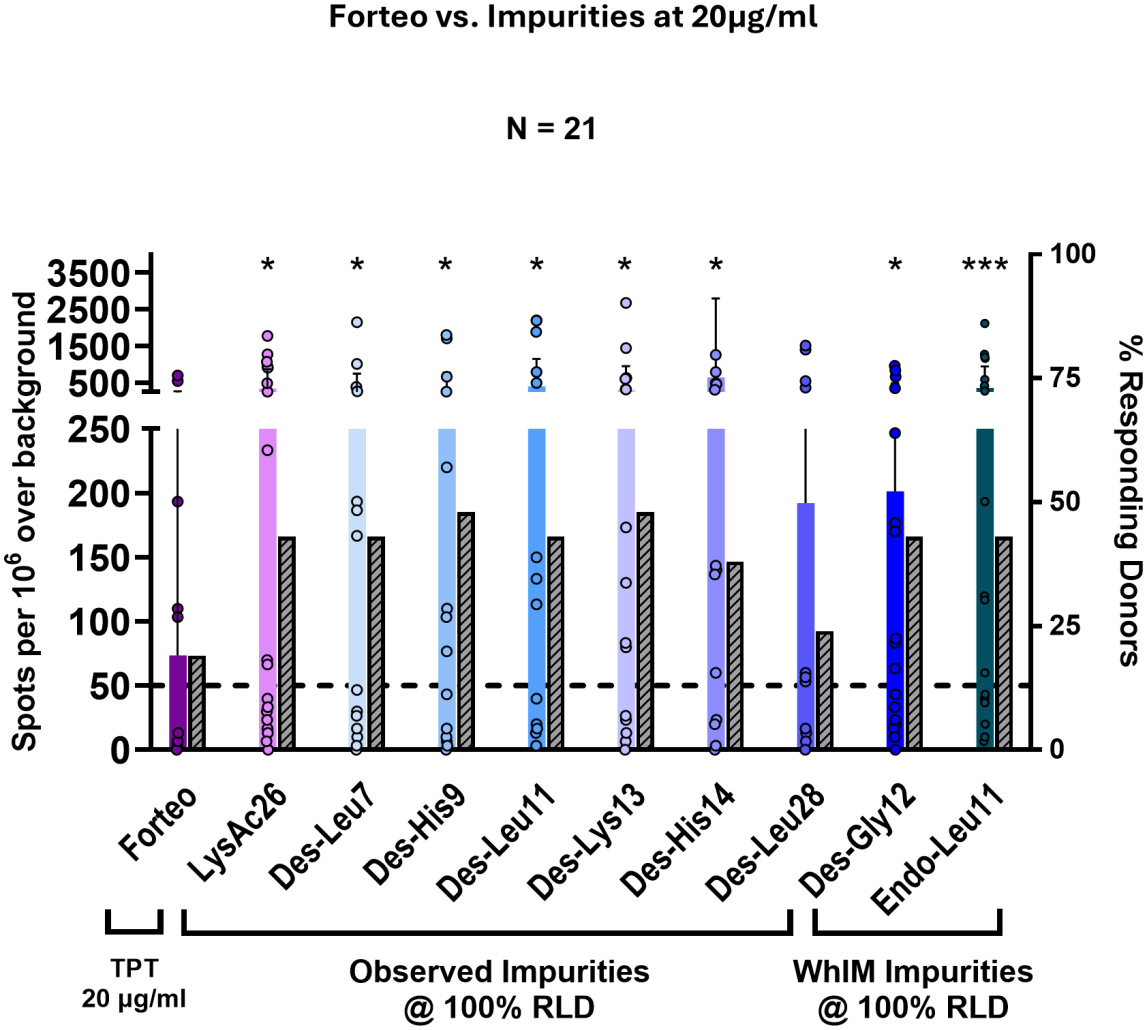
Donor PBMC responses to Forteo^®^ and individual impurities. Forteo^®^ and each impurity were tested in a 14-day naïve donor T-cell assay to measure immunogenicity by IFN-γ expression. Donor PBMC positivity included three criteria: (1) IFN-γ spot-forming cells (SFC) count > 50 per million cells, (2) stimulation index (SI) > 2, and (3) a statistical difference between media and peptide stimulation for SFC as determined by Student’s *t*-test (*p*<0.05). A total number of 21 donor PBMC samples were evaluated. The bar chart compares donor PBMC responses to the Forteo^®^ drug product versus each impurity tested at an equivalent concentration to the TPT RLD (20 µg/mL). Forteo^®^ and each impurity were evaluated at 20 µg/mL. The right-hand *y*-axis and grey bars show the percentage of responders (%) to each of the test peptides based on the positivity criteria. The left-hand *y*-axis and colored bars show the distribution of donor PBMC responses by the number of IFN-γ spot-forming cells (SFC) for the Forteo^®^ and the TPT impurities. Colored bars represent the mean values for each test article, with individual donor PBMC responses shown as circles. In the studies presented here, peptide impurities were reconstituted in RPMI-1640 cell culture media. See **Supplementary Figure S4** for a comparison of RPMI media versus Forteo^®^ formulation. Data normality was assessed using the Shapiro–Wilk test. As the data were not normally distributed, significant differences between Forteo^®^ and each impurity were determined using a paired Wilcoxon signed-rank test. Statistical significance is indicated by ^*^p ≤ 0.05 and ^***^p ≤ 0.001.

As shown in **Supplementary Figure S5**, when compared to impurity peptides reconstituted in RPMI-1640 cell culture media, no difference in the T-cell response, measured by IFN-γ secretion was observed when compared to impurity peptides from the same manufacturing lots reconstituted in our laboratory-made product formulation buffer. For this reason, the data presented were from studies where impurities have been reconstituted in RPMI-1640 culture medium. The immunogenicity of individual impurities was then compared to Forteo^®^, the TPT RLD, as shown in **Figure 7**.

In this case study, donor PBMC responses to the RLD were assessed to formulate the product (Forteo^®^, in this case) rather than the API peptide. Differences in immunogenicity between Forteo^®^ and the individual peptide impurities were assessed with two different approaches: (1) the frequency of PBMC donor that elicited T-cell responses (IFN-γ secretion) that were positive (SFC > 50 and SI > 2) within the study cohort (**Figure 7**, hashed grey bars; and (2) IFN-γ SFC distribution plot, which compared the mean distribution of the IFN-γ-secreting cells per million PBMCs of Forteo^®^ to the impurities for the donor PBMC cohort (**Figure 7**, colored bars showing the mean SFC count for each donor PBMC response, with individual donor samples represented by circles).

Each of the selected impurities elicited an IFN-γ response in a greater number of donors when compared to Forteo^®^ (**Figure 7**, hashed grey bars). Consistent with the increase in PBMC donor response rate, the mean expression of IFN-γ SFCs per million cells increased for each of the individual impurities when compared to the RLD product (**Figure 7**, colored bars and circles). These results suggested that eight of the nine selected impurities (the exception being Des-Leu28, the peptide with the highest JanusMatrix score) have a higher immunogenic risk potential compared to Forteo^®^, either due to less “human-like” content (lower JanusMatrix score) or the introduction of new T-cell epitopes (minimally different JanusMatrix score but higher epitope content). These impurities have the potential to enhance the immunogenicity risk of a generic TPT drug product if present in sufficient quantities in the final product. A summary of the *in silico* analysis is compared to the results of the naïve donor T-cell assay to make an overall assessment of risk in **Table 4** below. Additional examples of another set of TPT impurities with a loss of “human-like” content from independent studies can be found in **Supplementary Figure S3**.

**Table 4:** Overall risk based on *in silico* and *in vitro* results.

Peptide classification	Peptides	EpiMatrix score (immunogenicity potential)	JanusMatrix score (tolerance potential)	*In vitro* assay T-cell response rate (IFN-γ)	Overall risk
RLD	Forteo^®^	16.03	4.74	19%	Low
API	Teriparatide			4%–8.7%	
Impurities	DES_GLY12, ENDO-Leu11 and DES-LEU11	> 16.03 (high)	< 3.00 (low)	43%	High
	DES-HIS14, DES-LYS13, and LYS-AC26	> 16.03 (high)	3.00–4.74 (medium)	38%–48%	High
	DES-HIS9	10.00–16.03 (medium)	< 3.00 (low)	43%	High
	DES-LEU7	< 10.00 (low)	< 3.00 (low)	43%	Medium
	DES-LEU28	10.00–16.03 (medium)	> 4.74 (high)	24%	Low

*In silico* EpiMatrix (immunogenicity potential) and JanusMatrix (tolerance potential) scores were compared with the results of the *in vitro* naïve donor T-cell assay to provide an overall assessment of risk.

We note that impurity Lys-Ac26 was considered a nonbinder when evaluated in the HLA binding assays. Peptides evaluated in the IVIP T-cell assay are full-length peptides, while peptides tested in the HLA binding assay are shorter peptides, 12–20 amino acids in length (consistent with the length of Class II HLA presented peptides), with the impurity modification centered within the peptide. From the *in silico* analysis, impurity Lys-Ac26 has both higher predicted epitope content and reduced human homology compared to the TPT API, suggesting that this impurity would have greater immunogenic potential when compared to TPT (Forteo^®^), consistent with the results of the T-cell assay. Additional studies, such as three-dimensional peptide-MHC modeling, may clarify the discordance between these two results. The limited number of flanking amino acids (3) that are present at the N terminus could influence the peptide core binding resulting in the “no binding” classification Alternatively, this peptide could contain other process-related impurities that increase the response of a 14-day T-cell activation assay, although they would only be present in minute quantities in a synthetic peptide that is 95% pure.

Immune responses to the peptide impurities were lower when evaluated at a concentration of 0.2 µg/mL (1.0% of the API) compared to impurities evaluated at 20.0 µg/mL (see **Supplementary Figure S4**). Although this might suggest a lower risk of immunogenicity when impurities are present at the thresholds specified in the ANDA guidance, given that the TPT product is usually administered daily for 24 months or more, even an impurity with lower risk potential at specified limits could potentially exhibit a higher risk potential over months of repeat daily exposure.

**Figure 8:**
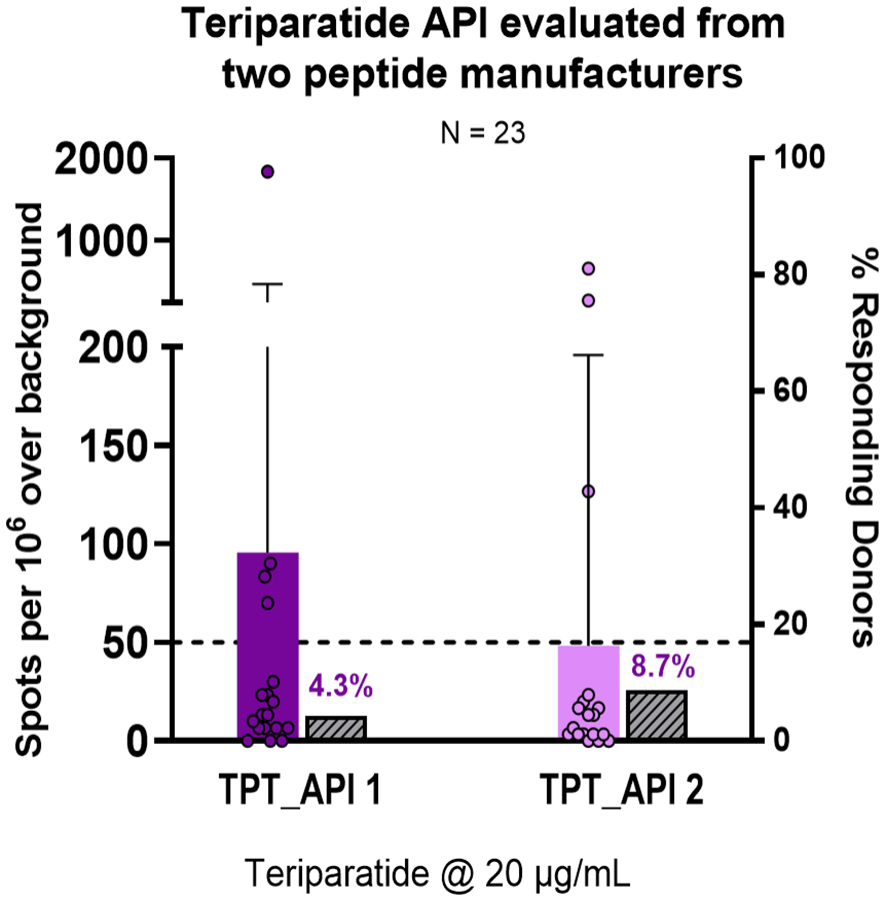
Donor PBMC responses to teriparatide API. Teriparatide API (unformulated) from two different peptide manufacturers was evaluated in a 14-day naïve donor T-cell assay to measure immunogenicity by IFN-γ expression. A total of 23 donor PBMC samples were evaluated. Donor PBMC sample positivity included three criteria: (1) IFN-γ spot-forming cells (SFC) count > 50 per million cells, (2) stimulation index (SI) > 2, and (3) a statistical difference between media and peptide stimulation for SFC as determined by Student’s *t*-test (*p*<0.05). The distribution of donor PBMC responses by number of IFN-γ SFC is shown for the TPT API peptides (purple bar), and the percentage of positive donors (that met positivity criteria) is shown in the adjacent grey bar for each API. The median IFN-γ SFC is indicated by the purple line within the quartiles. No significant differences were observed between the two TPT API sources using a paired Wilcoxon signed-rank test.

Since *in vitro* immune responses may be reduced when *in vitro* assays are performed in the presence of some drug formulations (see Roberts et al. (24)), we performed an *in vitro* T-cell assay using two separate TPT API peptides in cell culture medium (from two different API manufacturers), rather than the RLD. Donor PBMC responded similarly to the two API peptides (**Figure 8**). Minimal immune responses to the TPT API peptide were observed in T-cell assays performed with multiple donor PBMC samples, when considering both the percentage of responsive PBMC donors and the magnitude of the IFN-γ cytokine response, as was also observed for the Forteo^®^ drug product.

In summary, the *in vitro* T-cell assay results were consistent with the above (independent) *in silico* assessments in that they suggested that some impurities have an overall higher immunogenic risk potential compared to TPT. Three of the nine impurities (Des-Gly12, Endo-Leu11, and Des-Leu11) had higher EpiMatrix Scores than the TPT API (>16.03) and low JanusMatrix Scores (< 3.00), indicating the highest risk for immunogenicity (dark red shading in **Figure 5b**). Forty-three percent of donors responded to these highest risk impurities at equal concentration to the API. Three of the nine impurities (Des-His14, Des-Lys13, and Lys-Ac26) had higher EpiMatrix scores (> 16.03) and lower JanusMatrix Scores (3.00–4.74) than the TPT API, indicating a high risk for immunogenicity (light red shading in **Figure 5b**). Between 38% and 48% of donors responded to these high-risk impurities at equal concentration to the API. One impurity (Des-His9) had a high EpiMatrix score (10.00–16.03) and a low JanusMatrix score (< 3.00), indicating a high risk for immunogenicity (light red shading in **Figure 5b**). Forty-eight percent of donors responded to this high-risk impurity at equal concentration to the API. One impurity (Des-Leu7) had a low EpiMatrix score (< 10.00) and a low JanusMatrix score (< 3.00), indicating a medium risk for immunogenicity (white shading in **Figure 5b**). A total of 43% of donors responded to this medium risk impurity at equal concentration to the API. One impurity (Des-Leu28) had a high EpiMatrix score (> 10.00) and a higher JanusMatrix score (> 4.74) than TPT API, indicating a high potential for immunogenicity, which is likely to be offset by homology-induced tolerance (light green shading in **Figure 5b**). Only 24% of donors responded to this low-risk impurity at equal concentration to the API.

**Figure 9:**
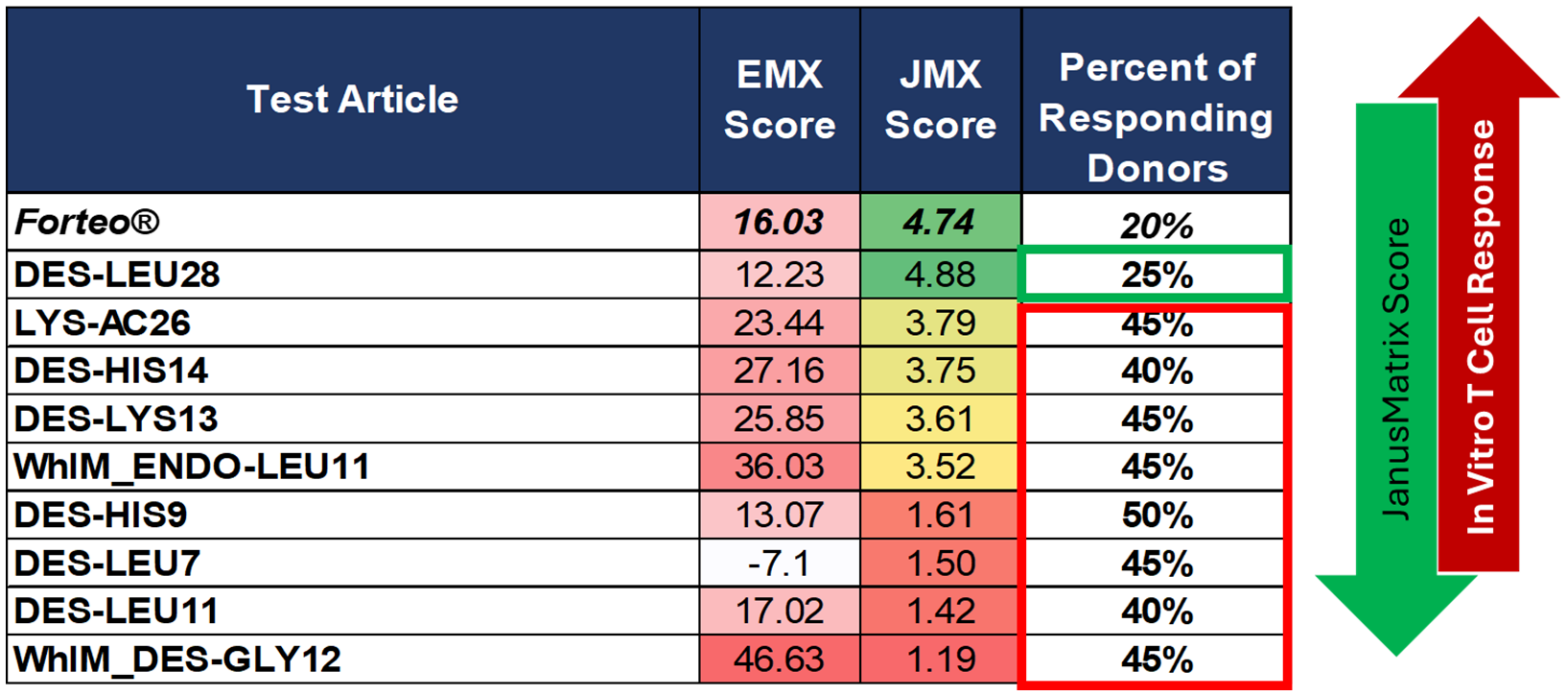
Summary of *in silico* scores and *in vitro* immunogenicity. Impurities are listed in order of descending JanusMatrix (human homology) score. A higher JanusMatrix score indicates greater cross-conservation with the human proteome and a higher potential for immune tolerance. The percentage of responding donor PBMC is also shown for each impurity, providing a measure of the *in vitro* immunogenicity observed in the naïve T-cell assay.

The assay results (T-cell assay, bystander assay) supported the observation that the promiscuous epitope identified in frame 5 (that is conserved with PTH and β-tubulin) may be tolerogenic. This potential tolerogenicity is based on the observation that any impurity peptide containing modifications in this region that changed the TCR face resulted in an increase in the number of donor PBMC samples that responded to the peptide, and increased the magnitude of the IFN-γ response as well. For example, immunogenic impurities Des-Leu7_TPT, Des-His9_TPT, and Des-Leu11_TPT all have modifications to TCR-facing residues in the purported Treg epitope sequence (frame 5), which reduced the JanusMatrix human homology relative to the API (see *in silico* scores listed in **Figure 9**). These results support the hypothesis that reduced cross-conservation with the human proteome or an increase in predicted T-cell epitope content may increase the immunogenicity of synthetic peptide impurities.

In addition to the seven synthetic TPT impurities selected for *in vitro* testing from the 34 identified by the FDA through internal studies, two theoretical impurities identified by the novel WhIM algorithm were evaluated in the *ex vivo* T-cell assay. These impurities, WhIM_ENDO-Leu11_TPT and WhIM_DES-Gly12_TPT, which were selected for their elevated EpiMatrix scores and reduced JanusMatrix scores relative to TPT, elicited a response in twofold more PBMC donors than the number that responded to TPT (**Figure 7**). Additionally, these two peptides elicited a greater number of IFN-γ spot-forming cells when compared to Forteo^®^ and the other TPT-derived impurities (**Figure 7**). These data also suggest that the WhIM algorithm could be used to prospectively identify peptide impurities with an increased risk potential in generic drug products. Results from WhIM could be used to flag potentially immunogenic peptides that would be likely to induce an immune response, for removal by improvements to the manufacturing process. TPT exhibits suppressive capabilities *in vitro*.

Having confirmed the promiscuous HLA binding of the epitope in frame 5 to multiple HLA-DR molecules using the class II HLA binding assay, we hypothesized that the suppressive capabilities of this epitope in the API could help explain the overall low clinical immunogenicity observed for TPT, as well as the reduced response seen in the *in vitro* assessment of T-cell responses. To examine this, we performed a tetanus toxin bystander suppression assay (TTBSA), which was adapted from a publication by Barbey et al. (25). This assay has been used to validate Treg epitopes identified in IgG and other human proteins (33).

Here, the assay was used to determine if the identified promiscuous epitope in frame 5 of TPT functions as a regulatory T-cell epitope. The core 9-mer sequence of the API with flanking residues, TPT_API_2–16_ (TPT_2–16_), was synthesized and evaluated in the TTBSA assay. The TTBSA assay was used to evaluate the putative Treg epitope TPT_2–16_ for its ability to suppress recall response to TT in PBMCs from TT-immune donors. Thus, to assess the suppressive capacity of the TPT_2–16_, CFSE-labeled human PBMCs were incubated with either media, TT alone, or TT+TPT_2–16_ at increasing concentrations for 7 days. Following 7-day incubation, cells were evaluated by flow cytometry for CD4 T-cell proliferation (CFSE dilution) and for activation status (CD4^+^CD25^high^). The immunosuppressive capacity of TPT_2–16_ was compared to the response to two well-characterized Tregitopes (289 and FV621) using the same donor PBMC in the assay, which is described in detail in De Groot et al. (33) and Miah et al. (34). Information on negative controls for the TTBSA is included in De Groot et al. (33). The detailed gating strategy for this assay is provided in **Supplementary Figure S6**.

**Figure 10:**
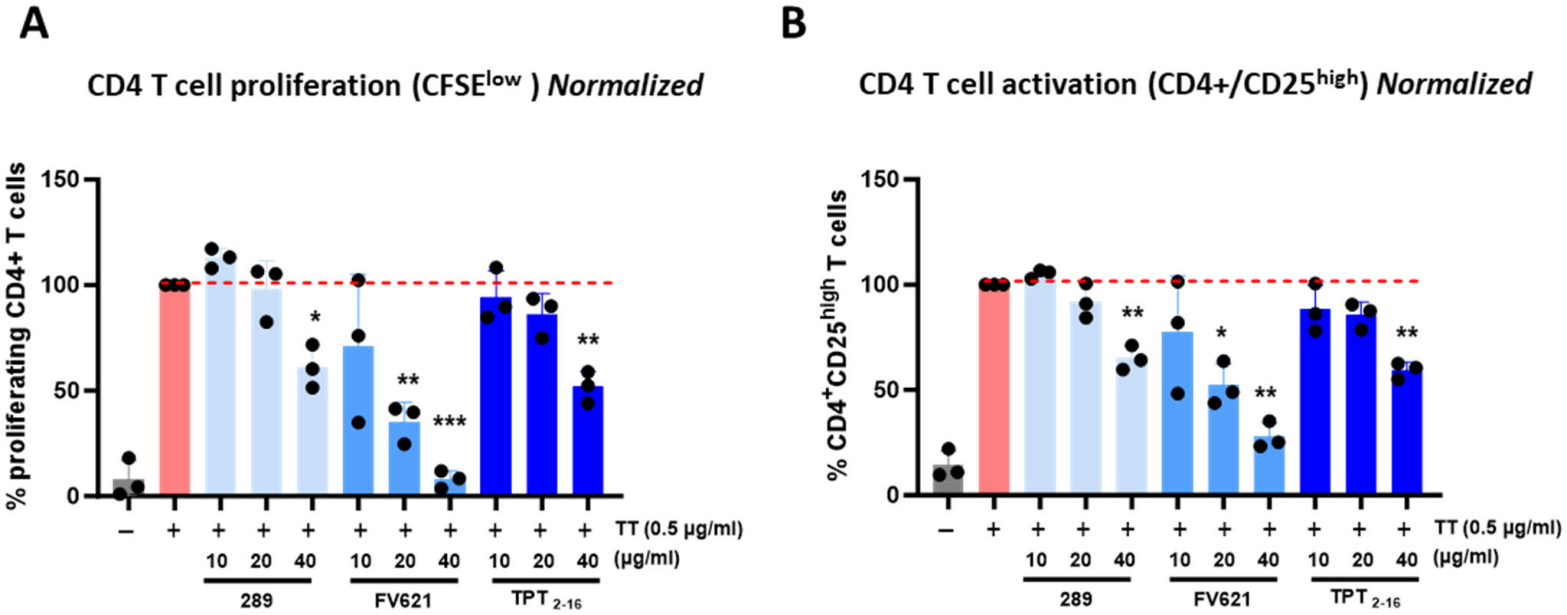
Representative flow cytometry histograms in TTBSA. Flow cytometry dot plots show results from a representative donor. The percentage of CD4 T-cell proliferation (CFSE low) is shown in **(A)**, and the percentage of CD4 T-cell activation (CD4^+^/CD25^+^) is shown in **(B)**. PBMC from this donor were stimulated with either media (left-most dot blot), TT alone (0.5 µg/mL), or TT (0.5 µg/mL) with increasing concentrations of TPT_2–16_ (10, 20, and 40 µg/mL). **(A)** Proliferation: representative dot plots show a 10-fold increase in CD4 T-cell proliferation upon TT stimulation, with suppression observed as TPT peptide concentrations increase. **(B)** Activation: CD4 T-cell activation increased fivefold with TT alone and decreased in a dose-dependent manner as TPT peptide concentrations increased. The red dotted line indicates the normalized percent of CD4 T-cell proliferation or activation in response to TT. Statistical significance of peptide costimulation versus TT only was determined using a two-tailed Welch’s *t*-test, with ^*^p<0.05, ^**^p <0.01, and ^***^p ≤ 0.001.

**Figure 10** provides the results of *in vitro* incubations and flow cytometry studies. This figure shows the results of experiments including **(a)** CD4 T-cell proliferation measurements and **(b)** CD4 T effector cell activation values in PBMC treated with TPT_2–16_ as compared to PBMC treated with either media alone or tetanus toxoid alone. CD4^+^ T cells treated with media alone (**Figure 10A**) showed minimal proliferation after 7 days of culture. Also shown in **Figure 10A**, CD4 T cells displayed a 10-fold increase in proliferation when stimulated with TT antigen alone. In contrast, proliferation to TT decreased in a dose-dependent manner when the cells were coincubated with increasing amounts of TPT_2–16_. Similarly, when the expression of CD25 (an activation marker) (35) on CD4^+^ T cells was measured in the same assay, cells treated with TT showed a fivefold increase in CD25^+^ expression compared to media alone (**Figure 10B**). CD4^+^ T cells exhibited a dose-dependent decrease in CD25 expression when cells were costimulated with TT plus the TPT_2–16_ peptide.

**Figure 11:**
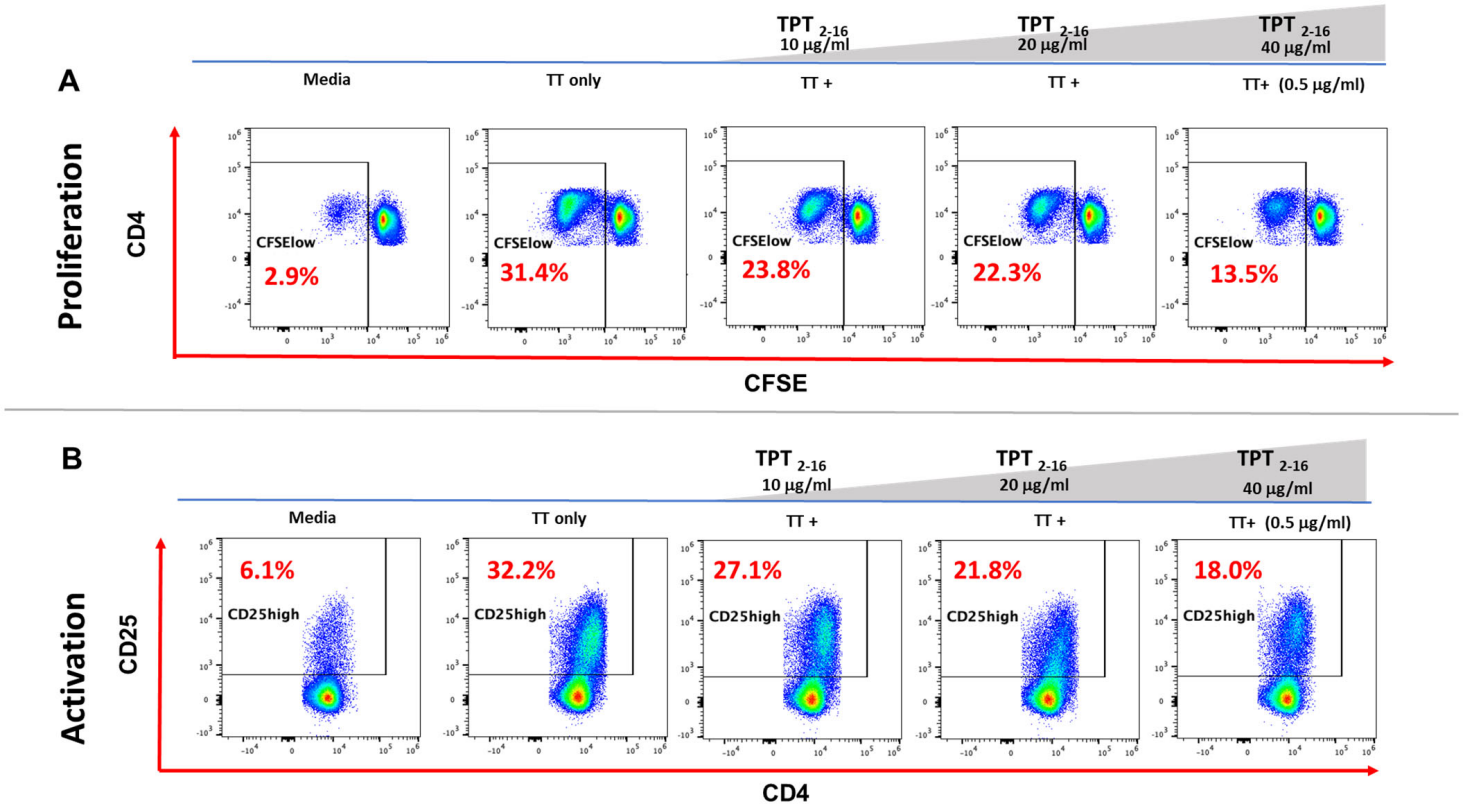
TPT_2–16_ suppresses TT-induced CD4^+^ memory cell proliferation and activation. **(A)** Comparison of tetanus toxoid-induced CD4 T-cell proliferation and activation in the presence of TPT _2–16_ and two Tregitopes (289 and FV621). Data were normalized to *TT-only* stimulation (100%) to compare the inhibitory effect of peptide costimulations on CD4 T-cell proliferation and activation. The graphs represent cumulative results from PBMC samples from three donors. The histogram shows that addition of TPT_2–16_ reduced proliferation relative to TT-only-treated cells at the 40-µg/mL concentration. The addition of TPT_2–16_ at 40 µg/mL reduced the proportion of activated (CD4^+^CD25^high^) T cells within the total CD4^+^ population compared to TT-treated cells **(B)**. TPT_2–16_ exhibited a significant suppressive effect on proliferation and activation, comparable to that of the previously identified Tregitopes 289 and FV621. Previously published TT bystander assays have been validated using “scrambled” negative control peptides; therefore, no negative control was included in this assay.

A normalized comparison of the TPT_2–16_-mediated suppression of CD4^+^ T-cell proliferation and activation for three donors is presented in **Figure 11**. Results for each individual donor are provided in **Supplementary Figure S7**. The TPT_2–16_ peptide suppressed memory response to TT at all doses, including at the highest peptide concentration of 40 µg/mL (*p* ≤ 0.01) in PBMC samples from all donors. The suppressive nature of TPT_2–16_ is comparable to that elicited by two previously identified and characterized Tregitopes, 289 (derived from human IgG) and FV621 (derived from factor V) (33) (**Figure 11**).

Collectively, these assays suggested that TPT contained a putative regulatory T-cell epitope in frame 5. This Treg epitope appeared to suppress T-effector (memory) response to a known antigen (TT in this case), so the presence of this tolerogenic epitope in the product may possess the capability to modulate the overall immunogenicity of TPT in clinical settings. The finding illustrates the importance of evaluating the type of T-cell that might respond to a given peptide drug, because disruption of a key regulatory T-cell epitope in impurities that were produced during the synthesis of the API might contribute to an increase in immunogenicity of the drug product in the patient population. Moreover, as the Treg epitope in TPT is a promiscuous HLA binder, impurities that impact this tolerogenic epitope may increase immunogenicity risk for a broad range of individuals in the treated population.

## Discussion

TPT has a history of having low immunogenicity in the clinic, with only 2.8% of treated patients developing antidrug antibodies after 12 months of treatment. Consistent with this, TPT induced IFN-γ production by responding T cells in fewer than 20% of donor cell preparations *ex vivo*. However, because different impurities can arise from different methods of peptide synthesis and production, it is important to understand whether any peptide-related impurities could contribute to the immunogenicity risk of generic TPT. Here, we performed an immunogenicity risk assessment for several peptide-related impurities identified in FDA studies on TPT, as well as two theoretical impurities predicted to be immunogenic by WhIM. The assessment included *in silico* analysis and two orthogonal *in vitro* studies. The results suggest that combining orthogonal *in silico* and *in vitro* assays can help identify impurities with the potential to increase the immunogenicity risk of TPT.

*In silico* comparison of TPT and the selected impurity sequences showed that sequences in the N-terminus (amino acids 1–18) of TPT contain a promiscuous T-cell epitope in frame 5 that binds to multiple class II HLA-DRB1 alleles *in vitro*. In contrast, amino acid sequences in the C-terminus of TPT (amino acids 18–34) exhibited lower HLA binding potential. Results from the HLA binding assays independently confirmed the *in silico* assessments.

Interestingly, analysis using the JanusMatrix *in silico* tool suggested that the promiscuous binding region of TPT (amino acids 1–18) had a high degree of homology with other human-genome, putative HLA-DR-restricted T-cell epitopes and may therefore induce tolerance rather than an effector T-cell response. The lower-than-expected *in vitro* responses, despite the presence of the promiscuous epitope, support the hypothesis that TPT contains a tolerated or actively tolerogenic T-cell epitope in the N-terminus of the sequence. The tolerogenic nature of this region was further supported by the results of the TTBSA shown in **Figure 11**, which demonstrated a reduction in the response to tetanus toxoid similar to that observed for two previously validated Treg epitopes (IgG-derived Tregitope 289 and FV-derived 621) in the BSA (26, 33). This observation may explain the relatively low incidence of immunogenicity of the drug product despite the high promiscuity of T-cell epitopes identified by *in silico* analysis of the TPT API peptide sequence. In support of this hypothesis, impurities that disrupted homology with self-epitopes in frame 5 (and were predicted to be immunogenic based on this loss of homology by the JanusMatrix tool) elicited responses in more donors (up to 45%) compared to Forteo^®^ in the CD4^+^ T-cell assays. These assays compared TPT to positive controls (IgG Tregitope 289 and FV Tregitope 621). Although negative controls for Tregitopes have been previously used in this assay (33), they were not included in the assay described here. For example, the impact of TPT on memory T-cell responses when combined with peptide impurities could be compared to the effects observed with negative controls, such as the FV peptides used in De Groot et al. (33) (**Figure 4**).

This finding of a putative Tregitope in the N-terminus of TPT has important implications for the immunogenicity of peptide impurities in generic drug products. Specifically, if the API peptide contains a Treg epitope that confers tolerance to the API itself, could this Tregitope also prevent unwanted immune responses to immunogenic impurities present in the drug product. It will be important to investigate whether the potential Treg epitope in therapeutic TPT can suppress *in vitro* immune responses to any immunogenic impurities in the final product. Combining the immunogenic epitopes with the tolerogenic epitope (see, for example, Liu et al. (36)) may help confirm whether the TPT Treg epitope can suppress immune responses to its own impurities, at least *in vitro*.

Assessment of the “model” peptide impurities investigated indicates that both the introduction of “new” epitope content and the “loss of human cross-conservation” were potential contributors to the immunogenicity of the impurities tested in the T-cell assay. For example, the results of the CD4^+^ T-cell assay (**Figures 7A, B**) show that IFN-γ responses to impurities Des-His9 and Des-Leu11 were similar to those observed for Des-His14 and Des-Lys13. On the surface, these results do not appear to align with the predicted immunogenicity shown in the quadrant plot (**Figure 5**). Des-Lys13 and Des-His14 have higher T-cell epitope content and slightly lower homology with the human proteome than the parent API, due to the introduction of new epitopes. Des-Leu11 and Des-His9, to which donors responded similarly, exhibited reduced cross-conservation with the human proteome. These observations suggest that either the loss of homology and/or introduction of new epitope content in peptide impurities can lead to increased immune responses (movement to the left or toward the top of the quadrant plot) compared to the parent API peptide.

In support of the concept that “foreign” epitopes may drive immunogenicity, while “tolerated or tolerogenic” epitopes may be less likely to drive immune response, studies by Howard et al. (38) showed differences in the immune responses to salmon calcitonin (SCT) and TPT in bone marrow–thymus–lymph node (BLT) immune-humanized mice. In contrast to responses observed to SCT, TPT had lower immunogenicity (as measured by activation of B cells) at multiple doses. The lower immunogenicity could be attributed to the foreign nature of the SCT “promiscuous epitope” or EpiBar region (24); however, the overall immune response to TPT was lower, despite similarity in immunogenicity scores (using EpiMatrix) between the two peptides. Increased numbers of long-term memory B cells and plasma cells were observed in SCT-treated mice as compared to TPT, which supported clinical observations of increased immunogenicity to SCT but not to TPT (37). B-cell maturation to long-term memory B cells and plasma cells is known to be suppressed by T-follicular regulatory T cells (38).

It is generally recommended that the assays to assess T-cell responses elicited by peptide impurities be performed using the same concentration as the API (formulated in saline), as this allows for direct comparison of relative immunogenicity. In the studies above, we observed that some of the impurities elicited more donors to induce higher IFN-γ responses relative to the API despite the 100-fold lower concentration. Moreover, we observed T-cell response to some impurities both when testing at 20 µg/mL and at the lower concentration of 0.2 µg/mL, which suggested that even when present at a lower concentration than the API, impurities may have the capacity to increase the overall immunogenic risk potential of a peptide drug product (**Supplementary Figures 3, 4**). Currently, it is considered to be more useful to assess immunogenicity at equal concentrations, i.e., testing the peptides at similar concentrations as the API.

Cell-based assays have inherent variability. When assessing single amino acid changes, the differences in responses may be subtle, which highlights the advantage of using a validated assay.

Here, in addition to the observed peptide impurities provided by the FDA, we generated a list of “potential” TPT impurities using the WhIM algorithm, which introduced iterative modifications and then performed immunogenicity risk assessment using the combination of EpiMatrix and JanusMatrix scores. This approach allowed us to select and generate higher-risk impurities to assess how the assays responded to higher-risk peptides. These impurities were predicted to be more immunogenic and less “human-like” than TPT, both *in silico*, and the observed *in vitro* T-cell response values were consistent with this prediction. Importantly, the responses observed in the HLA binding assay and the T-cell response assay aligned with the increased responses predicted by the *in silico* platform. The use of WhIM-generated impurities derived from the API peptide may be useful for generating “suitability controls” with both high and low risk of immunogenicity. These controls can be included in the T-cell assay alongside known positive controls (such as KLH) to show that the test was suitably sensitive for detecting responses to immunogenic peptides. In addition, impurity peptide controls that are independent of the manufacturers’ identified impurities can serve as useful control peptides that can be applied across multiple platforms and assessments.

At the time the T-cell assays were performed, the decision was made to compare the immune response of the drug product to the individual peptide impurities. Previous experience evaluating the salmon calcitonin RLD product, Miacalcin, indicated that a component of the formulation buffer was toxic to cells in culture or impacted the health of the cells such that response rates to Miacalcin, but not the salmon calcitonin API peptide, were lower than expected based on the known clinical immunogenicity data (24). Thus, to determine whether or not the formulation of the drug product may have had an impact on immune responses generated *in vitro*, we assayed T-cell responses to the formulation (recreated in our laboratory based on the ingredients listed in the label, see **Supplementary Figure S5**). We observed that the TPT formulation did not increase or decrease T-cell responses *in vitro*; thus, the drug-product to impurity comparisons are representative of API-to-impurity immunogenicity risk. This observation also suggests that the impact of formulation on the assays needs to be determined on a case-by-case basis.

In summary, these studies indicate that the relative risk of TPT, Forteo^®^, and generic peptide-related impurities can be assessed using orthogonal *in silico* and *in vitro* methods. Results from these or similar assays may be useful in designing studies to support submissions to regulatory agencies for generic drug products. Combining *in silico* and *in vitro* approaches to evaluate peptide-related impurity risk and inform the immunogenicity of candidate generic TPT is likely to improve the safety and availability of affordable generic products for clinical use.

## Data Availability

The raw data supporting the conclusions of this article will be made available by the authors, without undue reservation.

## Glossary

A*bbreviated New Drug Application (ANDA)*: Drug application to the US Food and Drug Administration (FDA) to manufacture and market a generic drug in the USA. The application is termed “abbreviated” since it does not require preclinical (animal) and clinical trials (human) to show safety and efficacy but requires the applicant to demonstrate bioequivalence to the brand-named drug.
Active Pharmaceutical Ingredient (API): The active ingredient in a drug product.
Antigen Presenting Cell (APC): A type of immune cell (e.g., dendritic cell, macrophage, and B cell) that binds peptide antigens via major histocompatibility complex (MHC) proteins on its surface and presents them to T cells.
ClustiMer: Tool for identifying epitope-rich polypeptides (with a high concentration of putative T-cell epitopes/HLA binders compared to other regions) within a given peptide or protein sequence.
Drug Product (DP): The finished dosage form of a drug, including the active pharmaceutical ingredient and inactive ingredients.
EpiMatrix (EMX): A proprietary algorithm developed at EpiVax to predict the likelihood of an amino acid sequence binding HLA.
EpiBar: *A single 9-mer frame predicted to bind to at least four different HLA-DR alleles.*
EpiMatrix Immunogenicity Score: The probability (represented by a *Z*-score) that a given peptide will bind to the HLA allele in question.
Human Leukocyte Antigen (HLA): Also referred to as major histocompatibility complex (MHC), a group of highly polymorphic proteins that display peptide antigens to T cells.
HLA ligand: Peptide predicted (*in silico*) or determined (*in vitro*) to bind to HLA.
JanusMatrix (JMX): Tool for predicting tolerance, putative Treg epitopes, and anti-self-immune responses.
Peptide-related impurity: A molecular variant of the peptide drug resulting from synthetic or storage (e.g., deletions, insertions, truncations, degradation, and/or aggregation).
Reference Listed Drug (RLD): The originator product for which a generic counterpart is made.
Tregitopes: Treg epitopes found in the IgG framework sequence of humans and other mammals that modulate antigen-specific effector T-cell responses. Non-IgG Tregitopes can be identified using JanusMatrix. Tolerogenic epitopes like Tregitopes are often extensively conserved with the human proteome.
T-cell receptor (TCR): The receptor that recognizes the HLA–peptide complex on APCs.
The Wiskott–Aldrich Syndrome protein homolog (WASH) complex: A protein complex essential for endosomal protein sorting, composed of five subunits, WASHC1 (WASH), FAM21 (WASHC2), WASHC3 (CCDC53), WASHC4 (KIAA1033), and WASHC5 (Strumpellin)
