# Supplementary material for: Immunogenicity risk assessment of peptide-related impurities identified in generic teriparatide products
Source: Front Immunol. 2025 Dec 8;16:1730346. doi: 10.3389/fimmu.2025.1730346 (PMC12722964; doi:10.3389/fimmu.2025.1730346)
Supplement: Supplementary file 1 [file DataSheet1.pdf]

Supplemental data for:

Immunogenicity Risk Assessment of Peptide-Related Impurities Identified in Generic Teriparatide Products

Aimee Mattei[1†], Brian J. Roberts [1†], Sandra Lelias[1], Shah Miah[1], Kristina E. Howard[2], James L. Weaver[2], Daniela Verthelyi[4], Eric S. Pang[3], Katie Edwards[5], Anne S. De Groot[1*]

[1] EpiVax Inc, 188 Valley Street, Suite 424, Providence, RI 02909, USA

[2] Division of Applied Regulatory Sciences, Office of Clinical Pharmacology, Office of Translational Sciences, Center for Drug Evaluation and Research, U.S. Food and Drug Administration, 10903 New Hampshire Ave, Silver Spring, MD 20993, USA

[3] Division of Therapeutic Performance I, Office of Research and Standards, Office of Generic Drugs, Center for Drug Evaluation and Research, U.S. Food and Drug Administration, 10903 New Hampshire Ave, Silver Spring, MD 20993, USA

[4] Division of Biotechnology Review and Research III, Office of Biotechnology Products, Office of Pharmaceutical Quality, Center for Drug Evaluation and Research, U.S. Food and Drug Administration, 10903 New Hampshire Ave, Silver Spring, MD 20993, USA

[5] CUBRC,4455 Genesse Street, Suite 106 Buffalo, NY 12445, USA

[†] Co-First authors

The purpose of these studies was to demonstrate the use of orthogonal methods to assess the immunogenicity risk of observed synthetic peptide impurities for teriparatide. Thirty-four FDA-identified impurities (referred to as 'observed' impurities) were evaluated in silico for their immunogenic potential using EpiMatrix and assessed for cross-conservation to the human proteome, or "humanness", using JanusMatrix (**see Supplemental Table 1** for the complete list of observed impurities). Seven impurities were selected from this list for further in vitro studies, including Class II HLA binding and the IVIP naïve T cell assay. In addition, two theoretical impurities identified with the "What if Machine", an algorithm that predicts potential impurities with immunogenic potential, were also included in the analysis. **Table 2** in the manuscript provides a list of all the selected impurities chosen for these in vitro studies.

For the IVIP naïve T cell assays PBMC from twenty-one donors were sourced from leukocyte reduction filters obtained from the Rhode Island Blood Center in Providence, RI. Males were represented at a slightly higher frequency than females (55% and 45% respectively). Overall, the average age of the donors was 50.4 years old, and the age range was 29-71 years old. The median age for the donor cohort was 53. Female donors were an average age of 44.9 years old with a range

of 29-57 years old. The median female age was 44.5 years old. Male donors were an average age of 55.5 years old with a range of 32-71 years old. The median male age was 58 years old. See **Supplemental Figure 1** PBMC donor cohort for demographics and HLA DRB1 represented.

Supplementary Figure 1: PBMC Donor Cohort Population HLA DRB1 Types and Demographics

Donor ID	Age	Gender	HLA DRB1	
1	32	Male	03:01	13:03
2	64	Female	04:01	13:01
3	47	Male	07:01	15:01
4	29	Female	04:05	07:01
5	54	Female	14:06	16:01
6	27	Female	03:01	03:01
7	56	Female	03:01	07:01
8	58	Male	08:01	11:04
9	62	Male	04:01	15:01
10	71	Male	11:02	13:01
11	41	Male	13:03	16:01
12	53	Male	11:01	15:01
13	46	Female	03:01	15:01
14	43	Female	15:01	15:01
15	45	Male	03:01	15:01
16	57	Female	13:01	13:03
17	70	Male	03:01	07:01
18	70	Male	07:01	10:01
19	37	Female	07:01	07:01
20	61	Male	03:01	07:01
21	36	Female	07:01	11:01

Donor Demographics			
	Female	Male	All
Percent	45%	55%	100%
Average Age	44.9	55.5	50.4
Median Age	44.5	58	53
Age Range	29-57	32-71	29-71

Global Supertype Population Coverage	
Population / Area	Class II HLA Coverage
East Asia	73.8%
Europe / North America	95.6%
Middle East	90.8%
North Africa	95.6%
South America / Other	77.4%
Sub Saharan Africa	94.9%
Average	88.0%
Standard Deviation	9.0%

Supplemental Figure 1: Donor Demographics. Males were slightly overrepresented in the cohort of donors. The average age of the donors was 50.4. A broad range of HLA DR alleles was represented in this cohort covering 88.0% of the alleles present in the global population.

Supplemental Figure 1 summarizes the HLA Types of the donors evaluated in this study. A broad range of HLA DR alleles was represented in the donor cohort with a projected population coverage of 88.0%. The population coverage statistics are based on MHC binding and T-cell restriction data developed by Bui et. al. (1) and made available on the Immune Epitope Database (IEDB) (2).

Supplemental Table 1: In silico results for 34 observed Teriparatide impurities

Impurity Type	Input Name	Input Sequence	EMX	JMX
**Baseline**	00_TERIPARATIDE_API	SVSEIQLMHNLGKHLNSMERVEWLRKKLQDVHNF	16.03	4.74
**Side Chain Modification:** Oxidation	01_MET-O8_TERIPARATIDE	SVSEIQLXHNLGKHLNSMERVEWLRKKLQDVHNF	14.81	4.47
	02_MET-O18_TERIPARATIDE_18X	SVSEIQLMHNLGKHLNSXERVEWLRKKLQDVHNF	16.01	4.58
	03_MET-OO18_TERIPARATIDE_18X	SVSEIQLMHNLGKHLNSXERVEWLRKKLQDVHNF	16.01	4.58
	04_MET-O8_18_TERIPARATIDE_8X_18X	SVSEIQLXHNLGKHLNSXERVEWLRKKLQDVHNF	14.79	4.32
	05_MET_O8_OO18_TERIPARATIDE_8X_18X	SVSEIQLXHNLGKHLNSXERVEWLRKKLQDVHNF	14.79	4.32
**Side Chain Modification:** Succinimide	06_ASU10_TERIPARATIDE_10X	SVSEIQLMHXLGKHLNSMERVEWLRKKLQDVHNF	9.72	4.19
	07_ASU16_TERIPARATIDE_16X	SVSEIQLMHNLGKHLXSMERVEWLRKKLQDVHNF	20.36	4.33
	08_ASU30_TERIPARATIDE_30X	SVSEIQLMHNLGKHLNSMERVEWLRKKLQXVHNF	22.04	3.86
	09_ASU30_TERIPARATIDE(1-30)_30X	SVSEIQLMHNLGKHLNSMERVEWLRKKLQX----	16.05	5.00
	10_ASU33_TERIPARATIDE_33X	SVSEIQLMHNLGKHLNSMERVEWLRKKLQDVHXF	16.03	4.74
	11_DHA17_TERIPARATIDE(1-30)_X17A	SVSEIQLMHNLGKHLNAMERVEWLRKKLQD----	16.16	4.82
**Side Chain Modification:** Lys Acetylation	12-1_LYS-AC13_TERIPARATIDE_13X	SVSEIQLMHNLGXHLNSMERVEWLRKKLQDVHNF	20.06	4.60
	13-1_LYS-AC26_TERIPARATIDE_26X	SVSEIQLMHNLGKHLNSMERVEWLRXKLQDVHNF	23.44	3.79
	14-1_LYS-AC27_TERIPARATIDE_27X	SVSEIQLMHNLGKHLNSMERVEWLRKXLQDVHNF	14.27	4.61
**Deletion(s)**	15_DES-ILE5_TERIPARATIDE	SVSE-QLMHNLGKHLNSMERVEWLRKKLQDVHNF	-0.54	3.09
	16_DES-LEU7_TERIPARATIDE	SVSEIQ-MHNLGKHLNSMERVEWLRKKLQDVHNF	-7.10	1.50
	17_DES-HIS9_TERIPARATIDE	SVSEIQLM-NLGKHLNSMERVEWLRKKLQDVHNF	13.07	1.61
	18_DES-LEU11_TERIPARATIDE	SVSEIQLMHN-GKHLNSMERVEWLRKKLQDVHNF	17.02	1.42
	19_DES-LYS13_TERIPARATIDE	SVSEIQLMHNLG-HLNSMERVEWLRKKLQDVHNF	25.85	3.61
	20_DES-HIS14_TERIPARATIDE	SVSEIQLMHNLGK-LNSMERVEWLRKKLQDVHNF	27.16	3.75
	21_DES-LEU15_TERIPARATIDE	SVSEIQLMHNLGKH-NSMERVEWLRKKLQDVHNF	14.55	4.67
	22_DES-LEU24_TERIPARATIDE	SVSEIQLMHNLGKHLNSMERVEW-RKKLQDVHNF	10.27	5.25
	23_DES-LEU28_TERIPARATIDE	SVSEIQLMHNLGKHLNSMERVEWLRKK-QDVHNF	12.23	4.88
	24_DES-GLN29ASP30_TERIPARATIDE	SVSEIQLMHNLGKHLNSMERVEWLRKKL--VHNF	23.10	4.14
**Duplication**	25_ENDO-SER18_19_TERIPARATIDE	SVSEIQLMHNLGKHLNSSSMERVEWLRKKLQDVHNF	17.87	4.48
**Truncation**	26_TERIPARATIDE(1-29)	SVSEIQLMHNLGKHLNSMERVEWLRKKLQ-----	16.97	5.00
	27_TERIPARATIDE(1-30)	SVSEIQLMHNLGKHLNSMERVEWLRKKLQD----	16.05	5.00
	28_TERIPARATIDE(1-32)	SVSEIQLMHNLGKHLNSMERVEWLRKKLQDVH--	17.88	4.74
	29_TERIPARATIDE(1-33)	SVSEIQLMHNLGKHLNSMERVEWLRKKLQDVHN-	16.95	4.74
	30_TERIPARATIDE(2-34)	-VSEIQLMHNLGKHLNSMERVEWLRKKLQDVHNF	16.95	4.74
	31_V_TERIPARATIDE(1-30)	VSVSEIQLMHNLGKHLNSMERVEWLRKKLQD----	15.12	5.00

Supplemental Table 1: In silico results for 34 observed Teriparatide impurities

Impurity Type	Input Name	Input Sequence	EMX	JMX
Extension	32_VR_TERIPARATIDE	VRSVSEIQLMHNLGKHLNSMERVEWLRKKLQDVHNF	16.14	4.60
N-term Modification	33_AC-TERIPARATIDE	SVSEIQLMHNLGKHLNSMERVEWLRKKLQDVHNF	16.03	4.74
	34_FA-TERIPARATIDE	SVSEIQLMHNLGKHLNSMERVEWLRKKLQDVHNF	16.03	4.74

Supplemental Table 1: In silico results for 34 observed Teriparatide impurities. EMX = EpiMatrix Score; JMX = JanusMatrix Human Homology Score. JMX Scores > 3.00 are considered elevated. Residues highlighted in red indicate differences in the impurity sequence compared to the API or proxy residues used in the unnatural amino acid substitution analysis. The red "X" represents a neutral placeholder. The red dash (-) indicates a deleted amino acid residue.

To further support the hypothesis that teriparatide impurities with modifications to the putative Treg epitope in frame 5, reducing the "humanness" of the impurity, can increase the immunogenic risk potential of the impurity compared to the teriparatide API peptide, data from impurity peptides from an independent study are provided. Each impurity was prospectively identified using the novel What If Machine (WhIM) tool. The WhIM-identified impurities were evaluated for their immunogenic risk potential (measure by IFNγ secretion) compared to the teriparatide API peptide using the same CD4+ T cell assay described in the methods. The results of this study further support the hypothesis that the tolerogenic nature of teriparatide is conferred by the promiscuous T cell epitope in frame 5. Single amino acid modifications to this 9-mer sequence are sufficient to disrupt tolerance and increase the immunogenicity of the impurities in vitro.

Supplemental Figure 2: HLA binding Data of TPT impurities (referenced in Figure 6)

HLA Binding for *DES-LEU7_TERIPARATIDE*

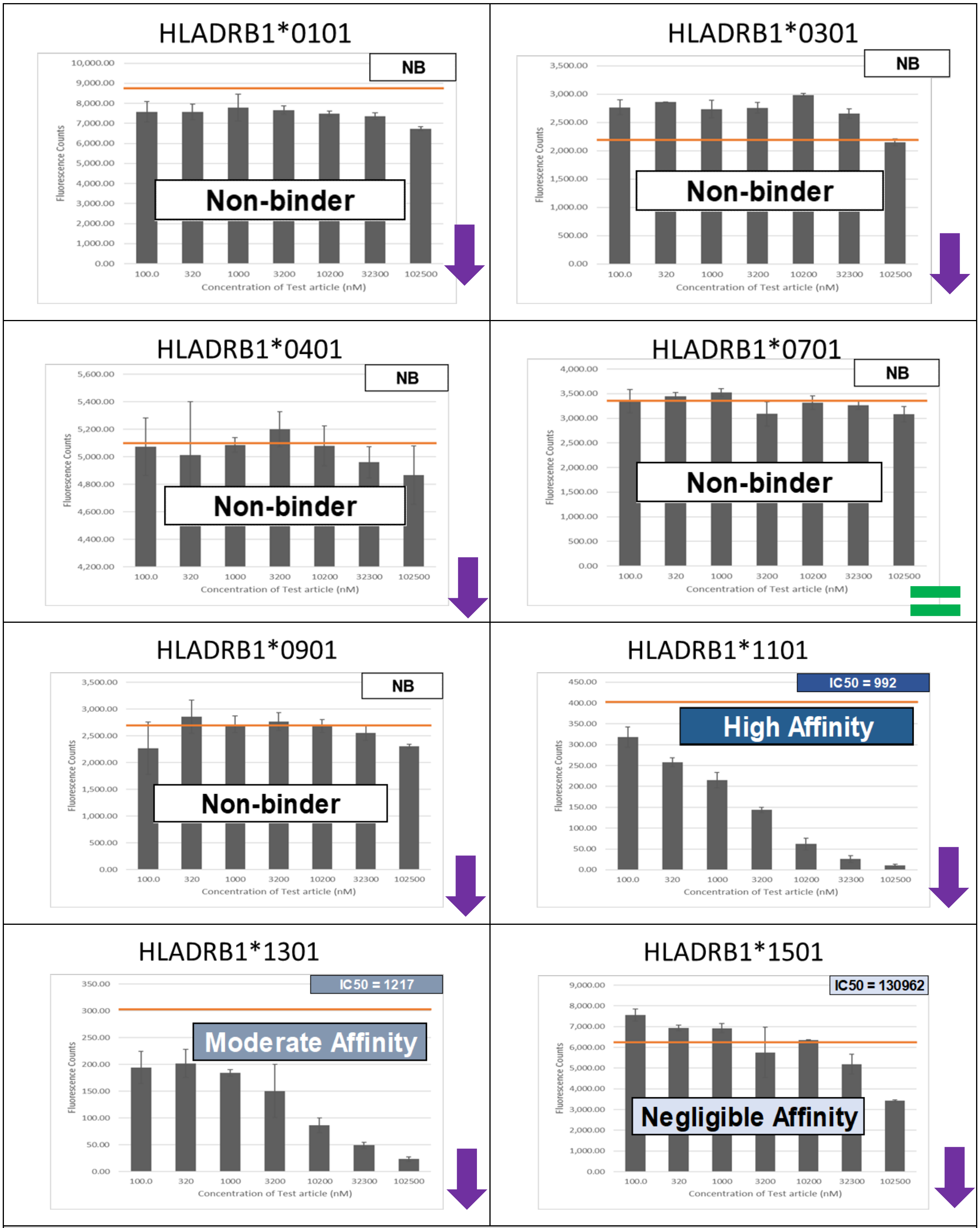

HLA Class II Binding Data for DES-LEU7_TERIPARATIDE

Blue shaded boxes indicate the IC50 values (nM) for the peptide for each tested allele; "NB" denotes a non-binder. The orange bar on each graph represents the maximum fluorescence value (no inhibition). A purple down arrow (↓) indicates a loss of observed binding affinity relative to Teriparatide and a red up arrow (↑) indicates an increase in observed binding affinity relative to Teriparatide. A green equal sign (=) indicates no change.

HLA binding for *DES-LEU28_TERIPARATIDE*

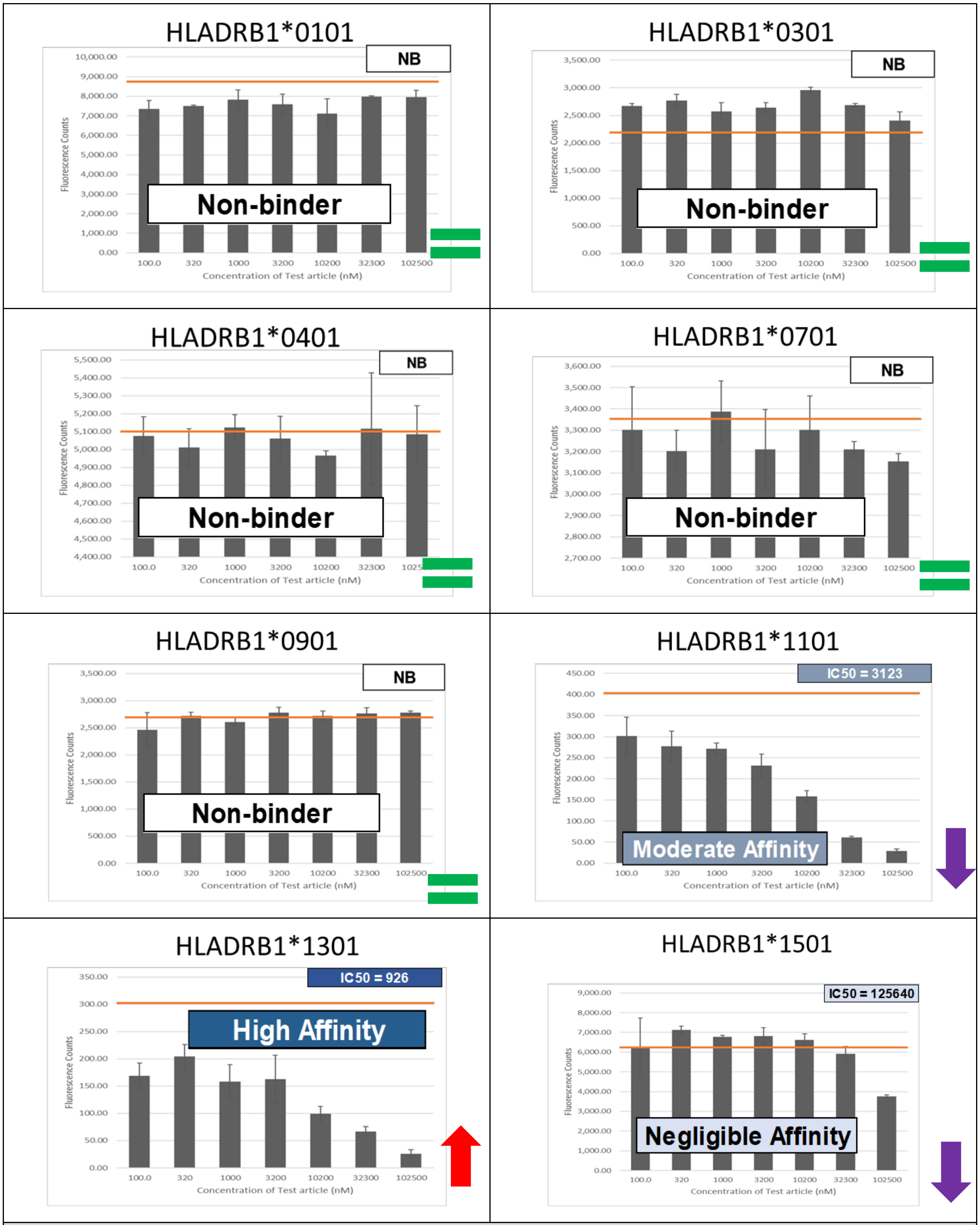

HLA Class II Binding Data for DES-LEU28_TERIPARATIDE

Blue shaded boxes indicate the IC50 values (nM) for the peptide for each tested allele; "NB" denotes a non-binder. The orange bar on each graph represents the maximum fluorescence value (no inhibition). A purple down arrow (↓) indicates a loss of observed binding affinity relative to Teriparatide and a red up arrow (↑) indicates an increase in observed binding affinity relative to Teriparatide. A green equal sign (=) indicates no change.

HLA binding for *LYS-AC26_TERIPARATIDE*.

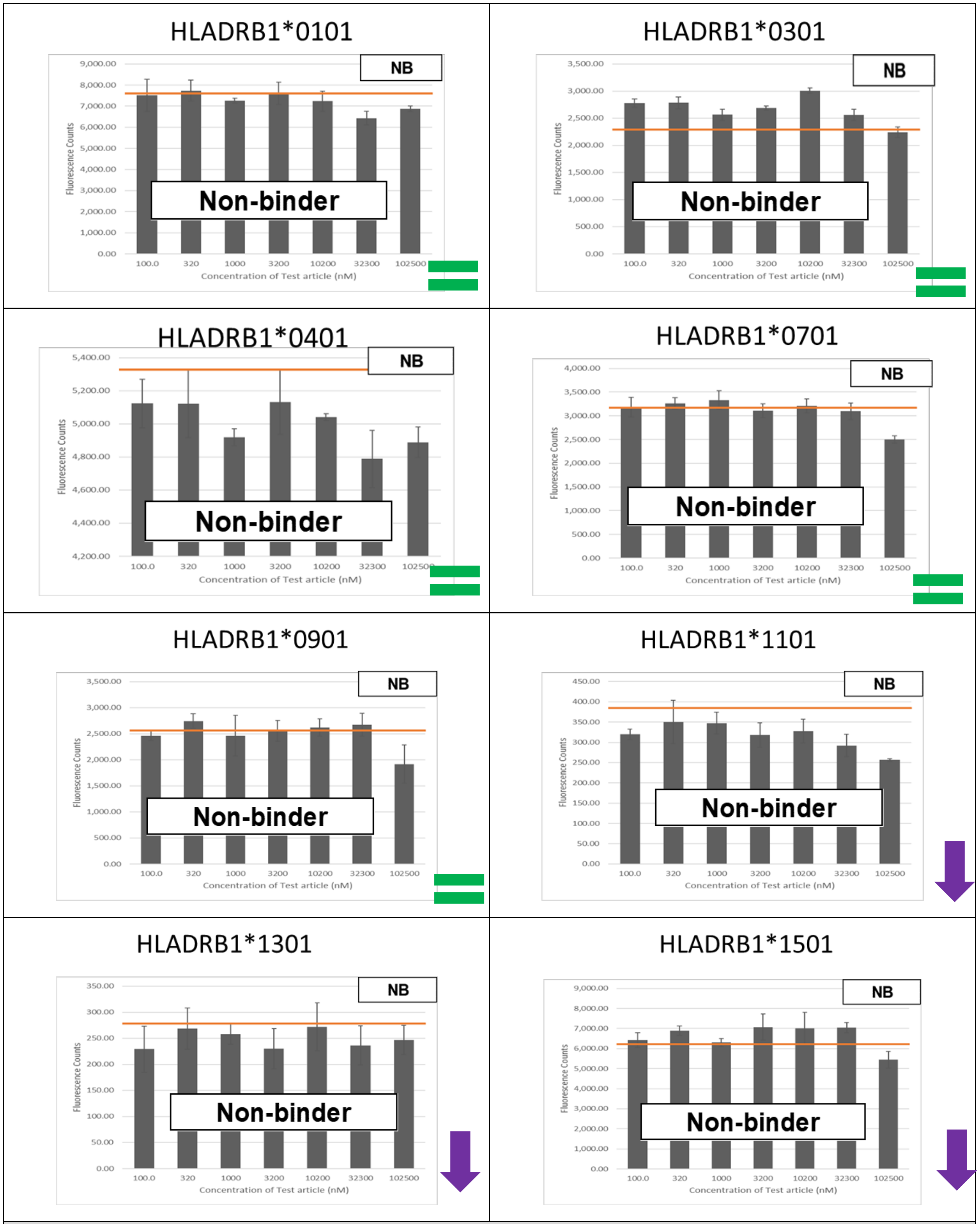

HLA Class II Binding Data for LYS-AC26_TERIPARATIDE

Blue shaded boxes indicate the IC50 values (nM) for the peptide for each tested allele; "NB" denotes a non-binder. The orange bar on each graph represents the maximum fluorescence value (no inhibition). A purple down arrow (↓) indicates a loss of observed binding affinity relative to Teriparatide and a red up arrow (↑) indicates an increase in observed binding affinity relative to Teriparatide. A green equal sign (=) indicates no change.

HLA Binding for *DES-HIS9_TERIPARATIDE*

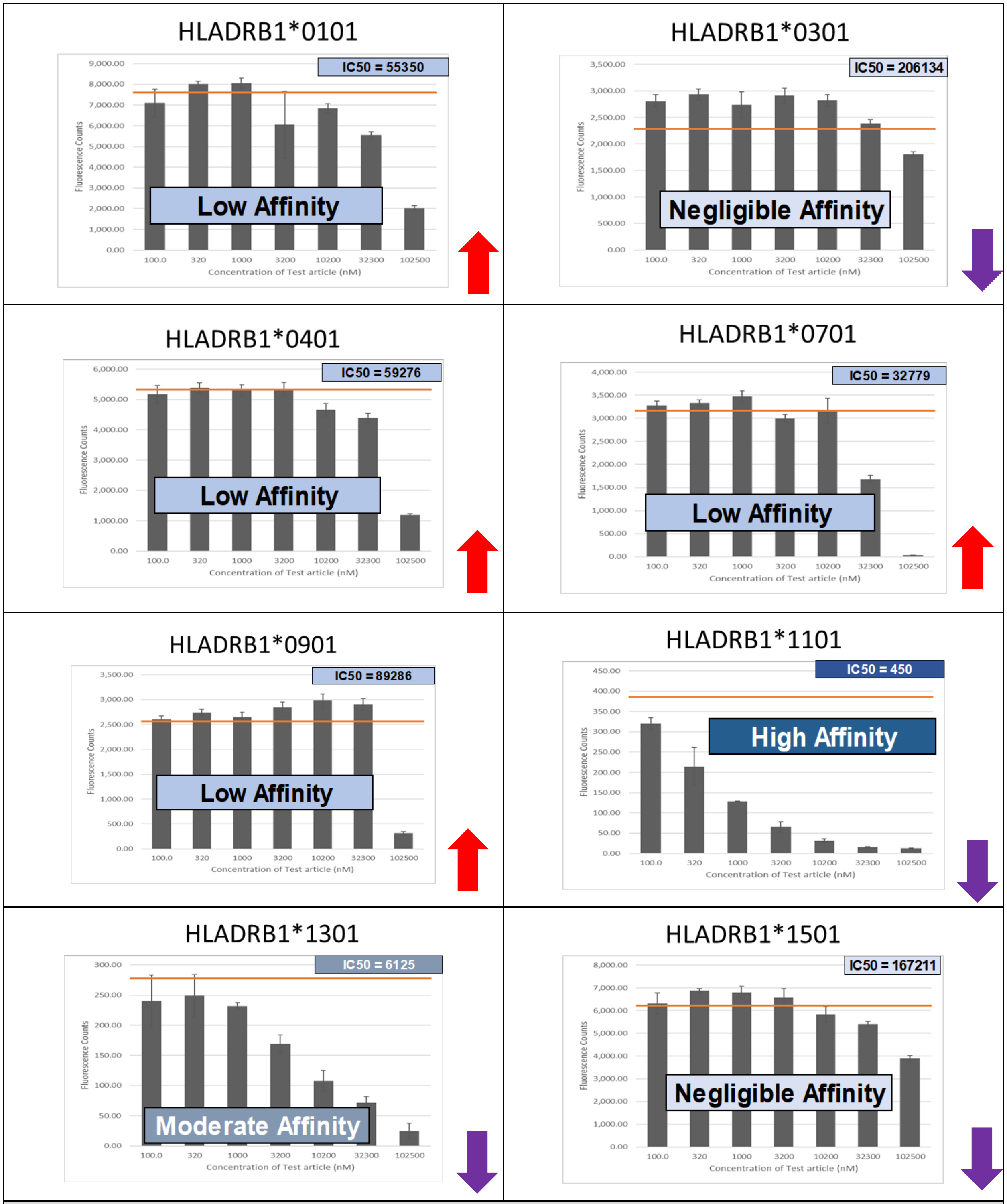

HLA Class II Binding Data for DES-HIS9_TERIPARATIDE
Blue shaded boxes indicate the IC50 values (nM) for the peptide for each tested allele; "NB" denotes a non-binder. The orange bar on each graph represents the maximum fluorescence value (no inhibition). A purple down arrow (↓) indicates a loss of observed binding affinity relative to Teriparatide and a red up arrow (↑) indicates an increase in observed binding affinity relative to Teriparatide. A green equal sign (=) indicates no change.

HLA binding for *DES-LYS13_TERIPARATIDE*

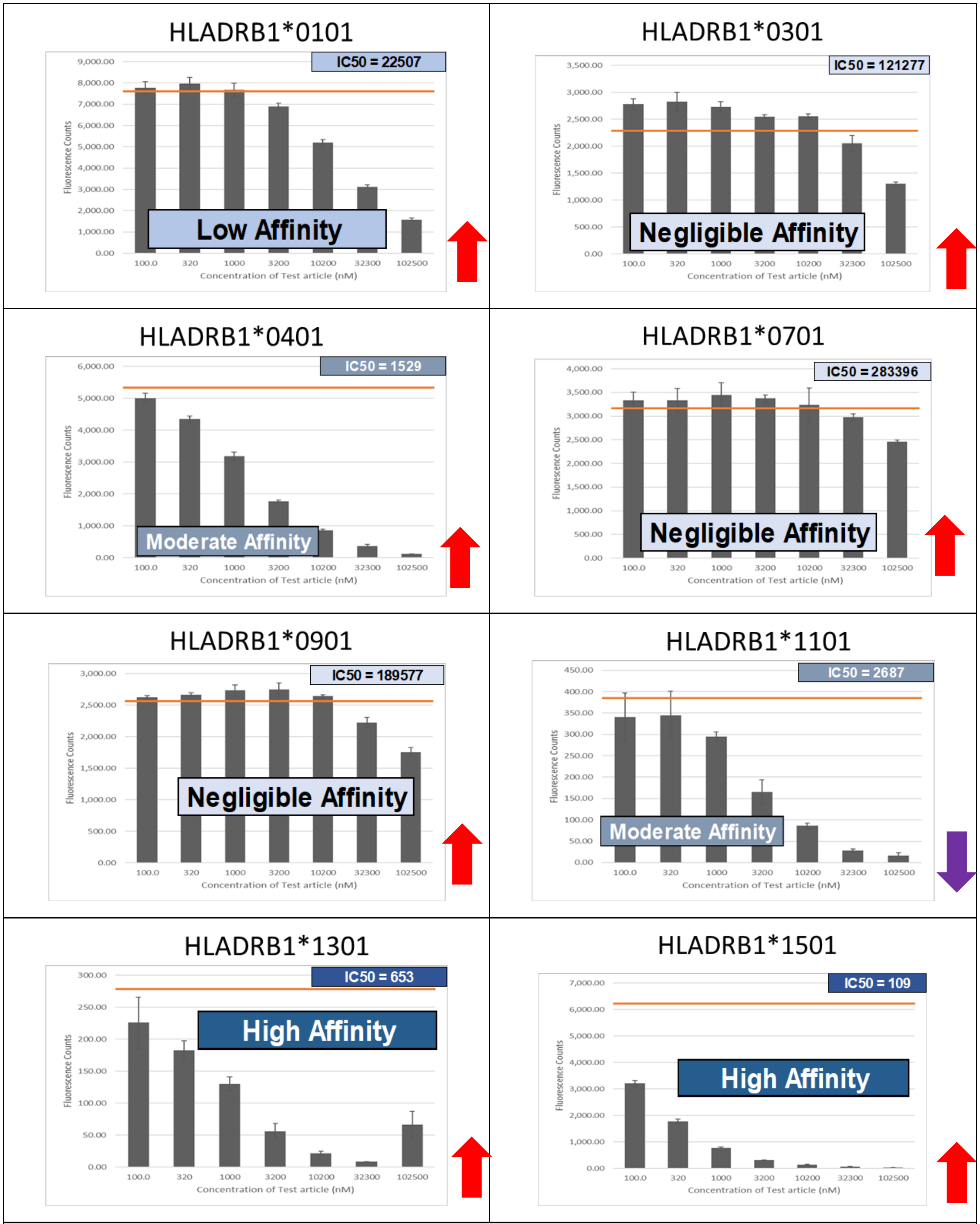

HLA Class II Binding Data for DES-LYS13_TERIPARATIDE
Blue shaded boxes indicate the IC50 values (nM) for the peptide for each tested allele; "NB" denotes a non-binder. The orange bar on each graph represents the maximum fluorescence value (no inhibition). A purple down arrow (↓) indicates a loss of observed binding affinity relative to Teriparatide and a red up arrow (↑) indicates an increase in observed binding affinity relative to Teriparatide. A green equal sign (=) indicates no change.

HLA binding for *DES-HIS14_TERIPARATIDE*

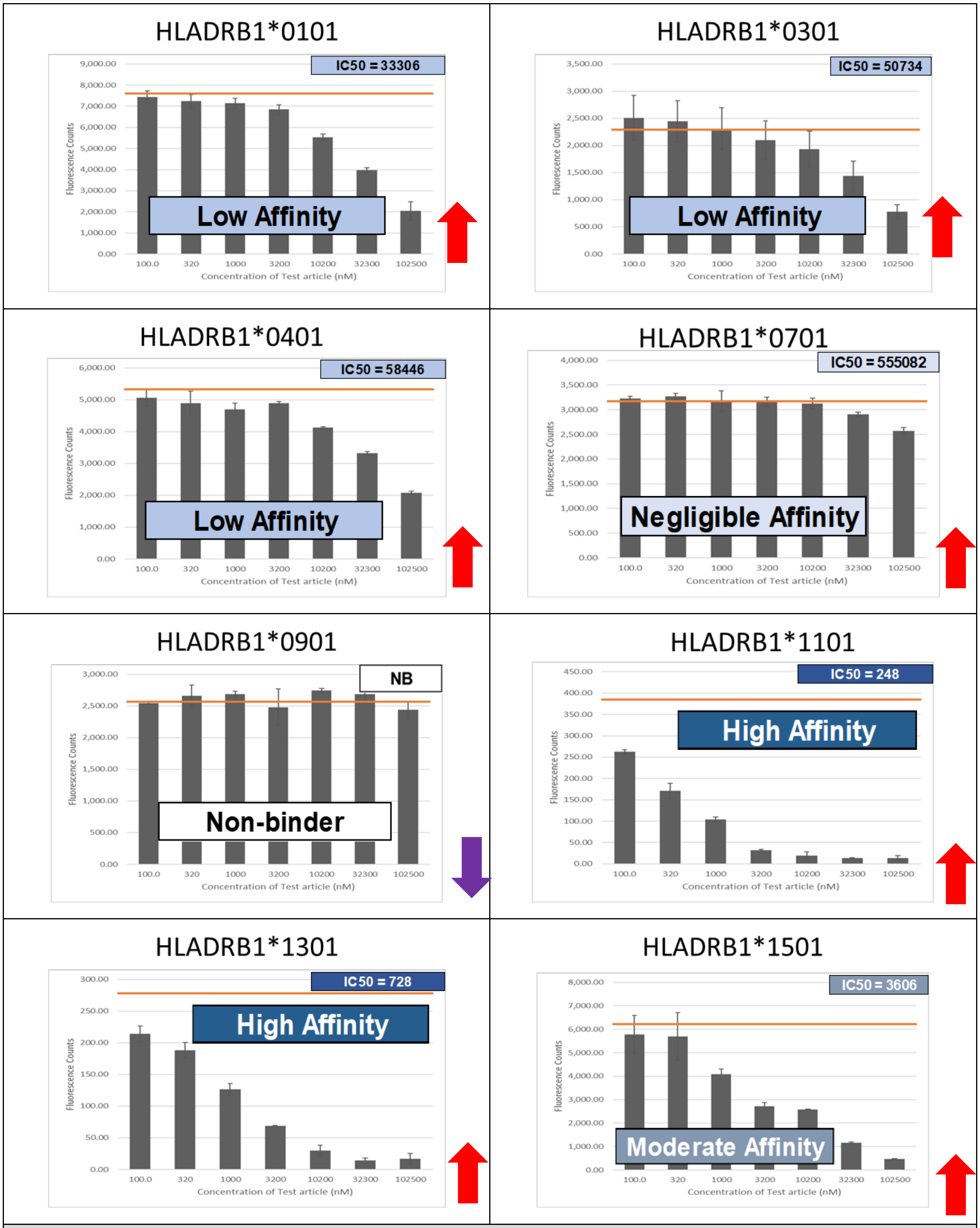

HLA Class II Binding Data for DES-HIS14_TERIPARATIDE

Blue shaded boxes indicate the IC50 values (nM) for the peptide for each tested allele; "NB" denotes a non-binder. The orange bar on each graph represents the maximum fluorescence value (no inhibition). A purple down arrow (↓) indicates a loss of observed binding affinity relative to Teriparatide and a red up arrow (↑) indicates an increase in observed binding affinity relative to Teriparatide. A green equal sign (=) indicates no change.

HLA binding for ***DES-LEU11_TERIPARATIDE***

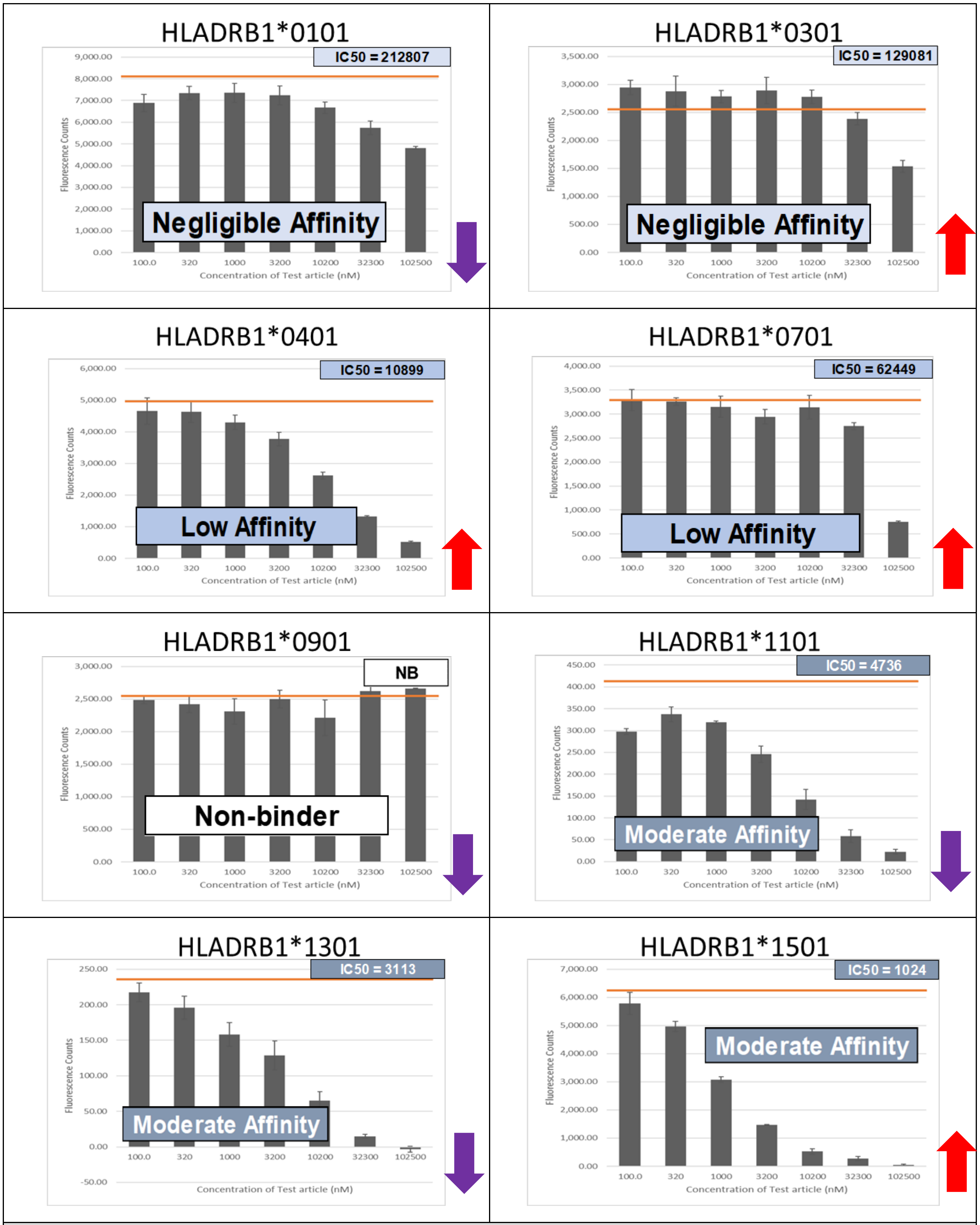

HLA Class II Binding Data for DES-LEU11_TERIPARATIDE

Blue shaded boxes indicate the IC50 values (nM) for the peptide for each tested allele; "NB" denotes a non-binder. The orange bar on each graph represents the maximum fluorescence value (no inhibition). A purple down arrow (↓) indicates a loss of observed binding affinity relative to Teriparatide and a red up arrow (↑) indicates an increase in observed binding affinity relative to Teriparatide. A green equal sign (=) indicates no change.

HLA binding for *WhIM_DES-GLY12_TERIPARATIDE*.

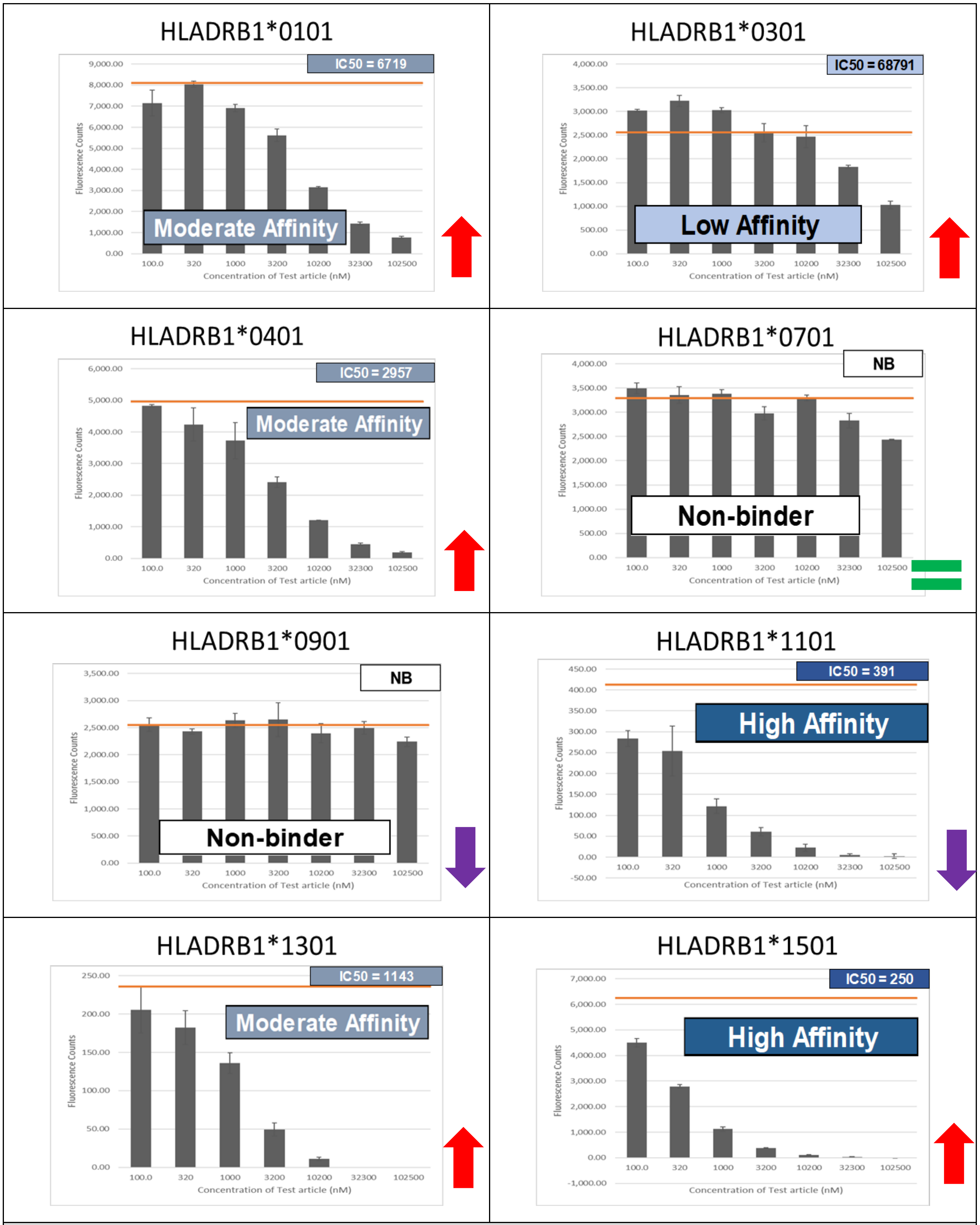

HLA Class II Binding Data for WhIM_Des-Gly12_TERIPARATIDE
Blue shaded boxes indicate the IC50 values (nM) for the peptide for each tested allele; "NB" denotes a non-binder. The orange bar on each graph represents the maximum fluorescence value (no inhibition). A purple down arrow (↓) indicates a loss of observed binding affinity relative to Teriparatide and a red up arrow (↑) indicates an increase in observed binding affinity relative to Teriparatide. A green equal sign (=) indicates no change.

HLA binding for *WhIM_ENDO-LEU11_TERIPARATIDE*.

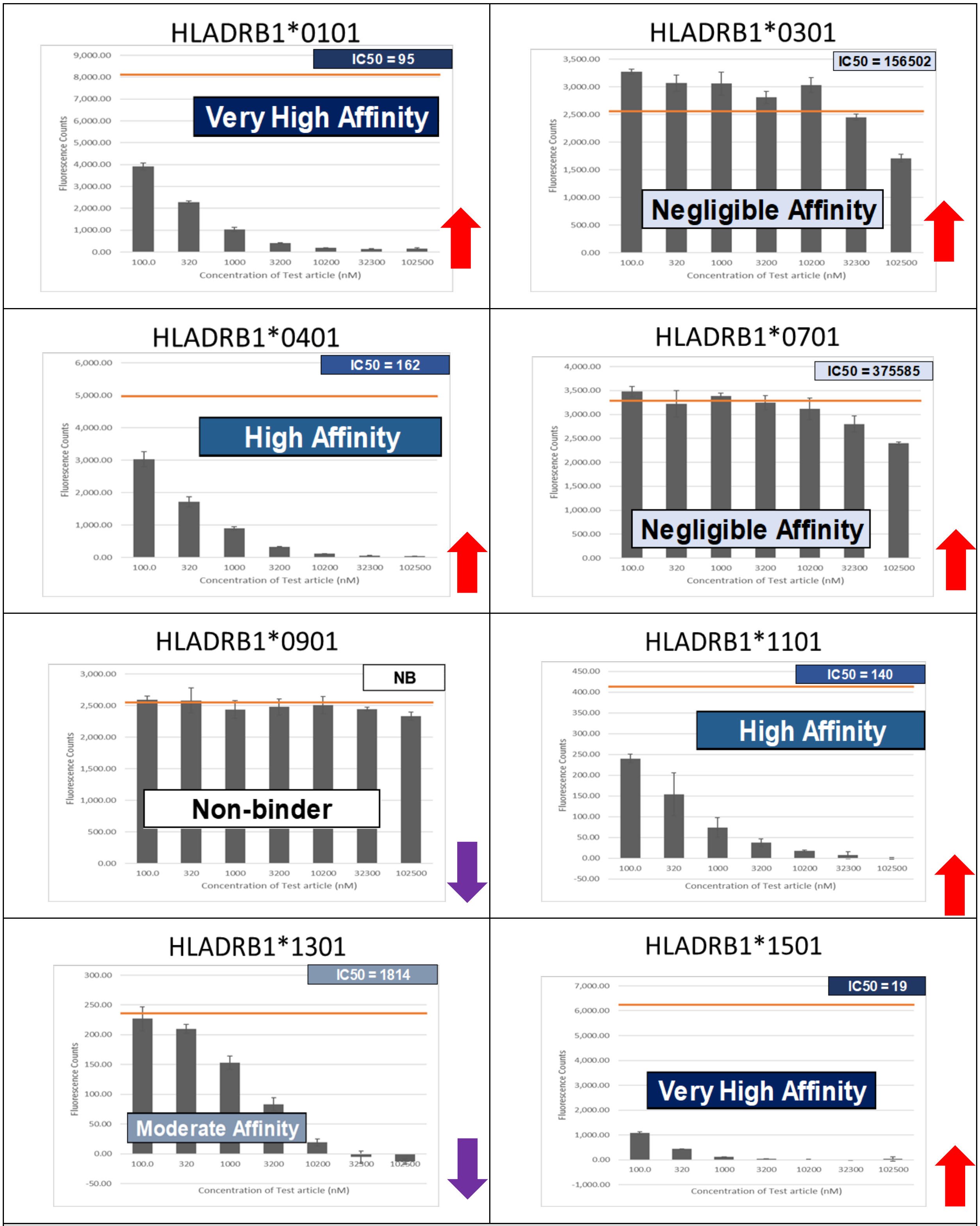

HLA Class II Binding Data for WhIM_Endo-Leu11_TERIPARATIDE

Blue shaded boxes indicate the IC50 values (nM) for the peptide for each tested allele; "NB" denotes a non-binder. The orange bar on each graph represents the maximum fluorescence value (no inhibition). A purple down arrow (↓) indicates a loss of observed binding affinity relative to Teriparatide and a red up arrow (↑) indicates an increase in observed binding affinity relative to Teriparatide. A green equal sign (=) indicates no change.

Supplemental Figure 3:

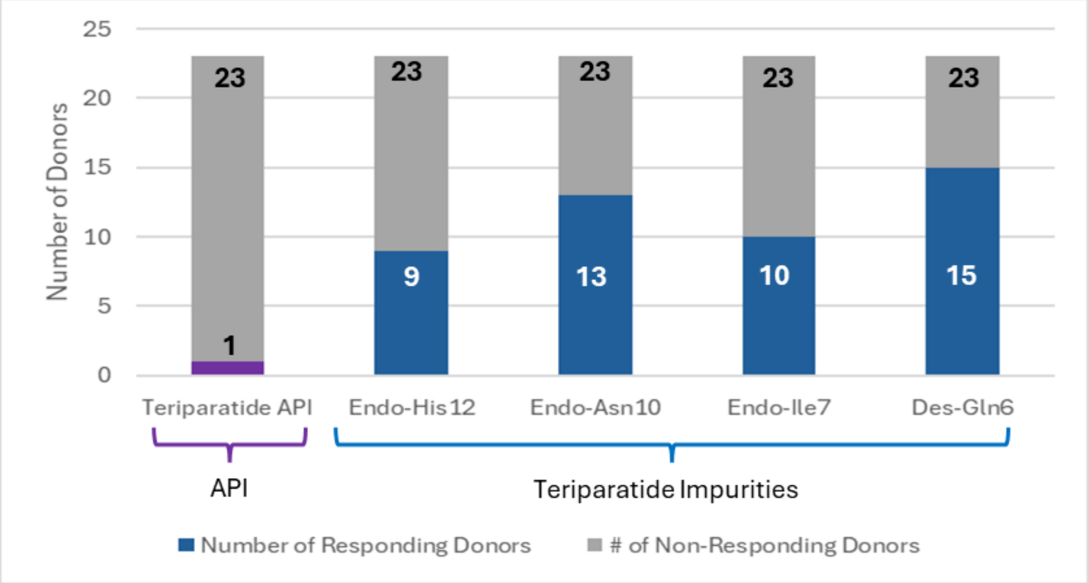

Name	Sequence	EMX Hits	EMX Score	JMX Score
**Teriparatide API**	**SVSEIQLMHNLGKHLNSMERVEWLRKKLQDVHNF**	**19**	**16.03**	**4.74**
Endo-His12	SVSEIQLMHNLHGKHLNSMERVEWLRKKLQDVHNF	22	24.17	0.82
Endo-Asn10	SVSEIQLMHNNLGKHLNSMERVEWLRKKLQDVHNF	19	20.16	1.68
Endo-Ile7	SVSEIQILMHNLGKHLNSMERVEWLRKKLQDVHNF	19	16.63	0.74
Des-Gln6	SVSEI-LMHNLGKHLNSMERVEWLRKKLQDVHNF	11	-0.64	1.27

Supplementary Figure 3. Additional data from a separate study showing that impurity modifications to the putative Treg epitope that led to a lower JanusMatrix score (loss of "humanness") increase the donor PBMC sample response rate in a cohort of 23 donors.

While regulatory reviewers prefer to see impurities evaluated at an equal concentration to API in T cell assays, we sought to determine if the concentration of the impurities impacts their immunogenic potential relative to Forteo®, the RLD. We evaluated the immunogenic risk potential of each impurity at 0.2 µg/ml, 1.0% of the concentration selected for TPT in Forteo®, close to the lower limit specified in the ANDA guidance (**Supplemental Figure 4**), using the same CD4+ T cell assay described in the methods. As seen below, the number of donors responding to the impurity is greatly reduced when the impurity is evaluated at a lower concentration, as compared to donors exposed to the impurity at the same concentration as the API to the API, even though some donors still do respond (**Figure 7**). It should be noted that at 0.2 µg/ml, the micromolar concentration of each peptide evaluated here is ≤ 0.05µM (**Table 2**), below the current recommendation that all peptides be evaluated at 0.5 µM. Use of impurity concentrations below 0.5 µM reduce the likelihood of generating a T cell response in PBMC, and could result in an underestimation of the immunogenic risk of a given peptide API or impurity.

Supplemental Figure 4: Donor PBMC responses to Forteo® and individual TPT Impurities at 0.2 µg/ml (1% of TPT RLD)

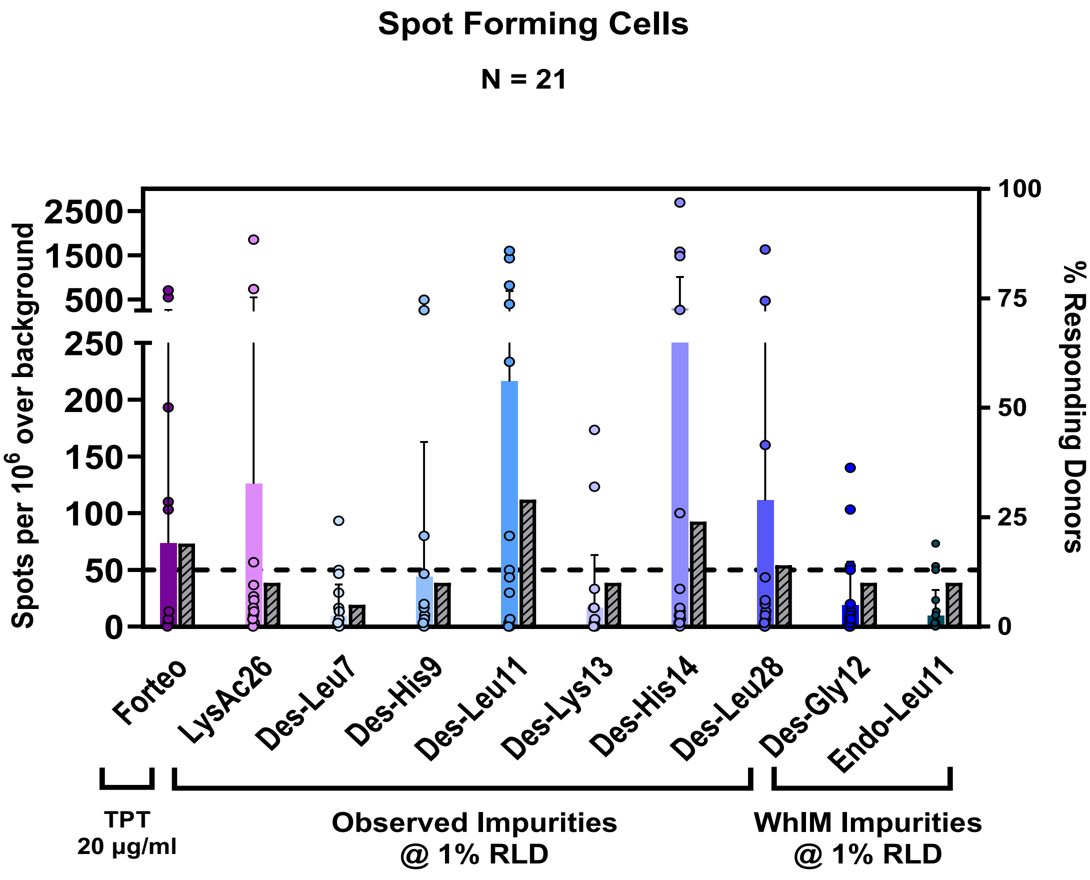

Donor PBMC responses to Forteo® and individual impurities. Teriparatide in Forteo and each impurity were tested in a 14-day naïve donor T cell assay to measure immunogenicity by expression of IFNγ. Donor PBMC positivity included three criteria: (1) IFNγ spot forming cells (SFC) count >50 per million cells, (2) Stimulation Index (SI) >2 and (3) a statistical difference between media and peptide stimulation for SFC as determined by Student t-test ($p < 0.05$). The total number of donor PBMC evaluated was 21. The bar chart provides a comparison of donor PBMC samples responding to Forteo, versus each impurity tested at 0.2 µg/ml (1% of the TPT API in the RLD). *The righthand y-axis* shows the percentage of responders (gray hashed bars) to each of the test peptides based on positivity criteria. *The lefthand y-axis* shows the mean number of IFNγ spot forming cells (SFC) for the Forteo and TPT impurities (colored bars) with individual donors represented by circles.

Supplemental Figure 5: Impact of product formulation on immunogenicity

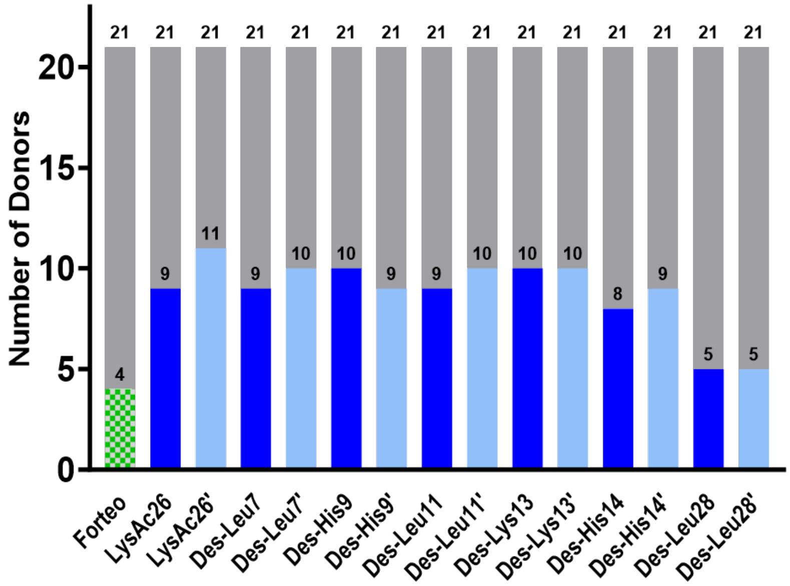

Supplemental Figure 5. Impact of Product Formulation on in vitro Immunogenicity. To determine if product formulation impacts immunogenicity in vitro, impurity peptides were reconstituted in cell culture media (dark blue bars) or with the Forteo® formula (light blue bars). No significant difference in donor response was observed between the two diluents. Data presented here is for peptides tested at 20.0µg/ml, identical to the concentration of teriparatide in Forteo that was evaluated, similar results were obtained for peptides tested at 1% (data not shown).

Supplemental Figure 6: TTBSA Flow Cytometry Gating Strategy

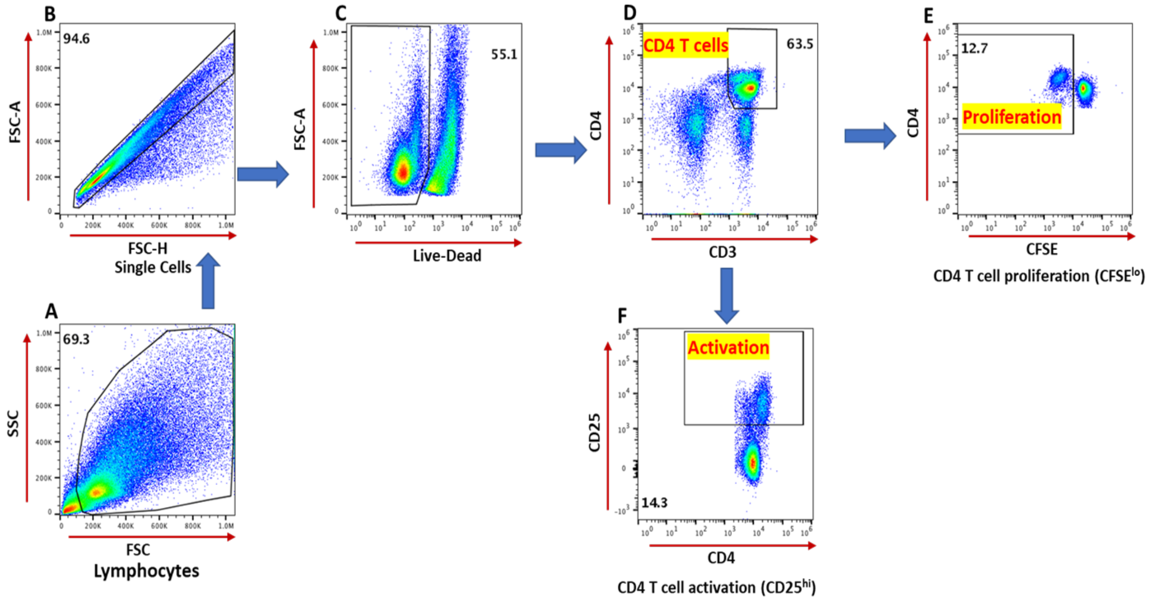

Supplemental Figure6. Representative gating strategy of the Lymphocytes population (panel A) used to identify CD4 + T cell proliferation (CFSE low)(panel E) and CD4+ T cell activation (CD4+/CD25 hi) (panel F) populations. The sequential gating strategy is illustrated by blue arrows.

Supplemental Figure 7: $TPT_{2\text{-}16}$ Suppresses TT-induced CD4+ Memory Cell Proliferation and Activation in donor PBMCs

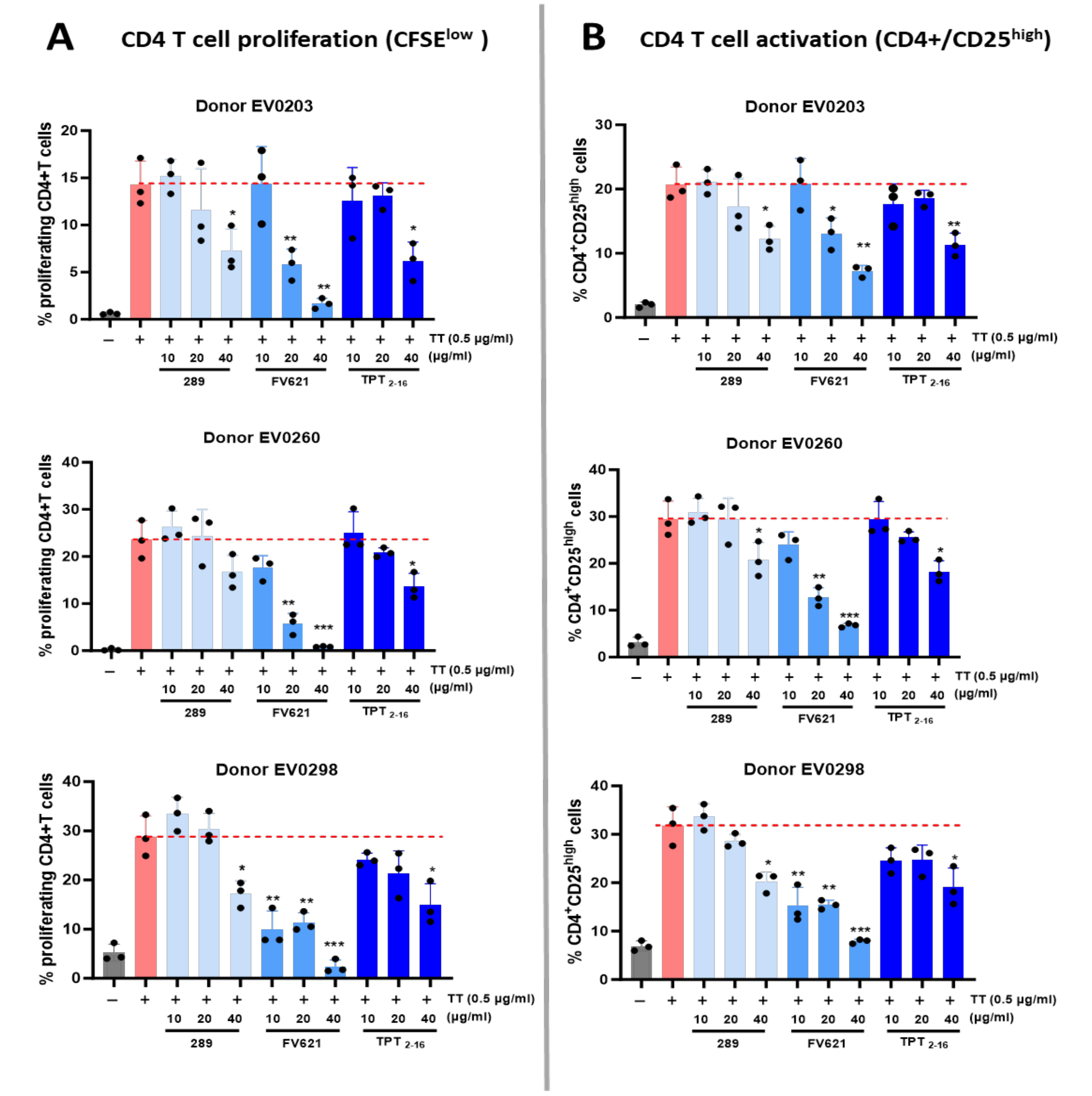

Supplemental Figure 7. Comparison of Tetanus Toxoid-induced CD4 T cell proliferation and activation response by costimulation with $TPT_{2\text{-}16}$ peptide and two Tregitopes (289 and FV621). For each donor the TT-only stimulation exhibited an increase in CD4 T cell proliferation (A) or percentage of $CD4^{+}CD25^{high}$ T cells (B) as compared to media-treated cells. (A) The costimulation with $TPT_{2\text{-}16}$ reduced proliferation relative to Tetanus Toxoid-treated cells for each donor at the 40µg/ml concentration. (B) Also, the addition of $TPT_{2\text{-}16}$ reduced the percentage of $CD4^{+}CD25^{high}$ T cells relative to Tetanus Toxoid-treated cells for each donor at the 40µg/ml concentration. The TPT $_{2\text{-}16}$ exhibited a significant suppressive effect on proliferation and activation comparable to that of Tregitope 289 and FV621. The **red dotted line** shows the percentage of cells proliferating in response to TT only. Significant differences (non-parametric Student's t test) between the co-stimulated peptide versus TT-only indicated as *p=<0.05, **p=<0.01, and ***p=<0.001.

References for Supplemental Information

1. Bui H-H, Sidney J, Dinh K, Southwood S, Newman MJ, Sette A. Predicting population coverage of T-cell epitope-based diagnostics and vaccines. *BMC Bioinformatics* (2006) **7**:153. doi:10.1186/1471-2105-7-153

2. Greenbaum J, Sidney J, Chung J, Brander C, Peters B, Sette A. Functional classification of class II human leukocyte antigen (HLA) molecules reveals seven different supertypes and a surprising degree of repertoire sharing across supertypes. *Immunogenetics* (2011) **63**:325–335. doi:10.1007/s00251-011-0513-0
